# Supplementary material for: Structural determinants of inequalities in untreated dental caries in the Global Burden of Disease Study
Source: PLoS One. 2025 Jun 3;20(6):e0325138. doi: 10.1371/journal.pone.0325138 (PMC12132969; doi:10.1371/journal.pone.0325138)
Supplement: S1 Appendix A — (DOCX) [file pone.0325138.s001.docx]

| Countries | Gov 2000 | Gov  2010 | Gov 2019 | Health  expenditure  2000 | Health  expenditure  2010 | Health  expenditure  2019 | SDI  2000 | SDI 2010 | SDI 2019 | % % Women Parliament 2000 | % % Women Parliament 2010 | % Women Parliament 2019 | GDP  2000 | GDP 2010 | GDP 2019 |
| --- | --- | --- | --- | --- | --- | --- | --- | --- | --- | --- | --- | --- | --- | --- | --- |
| Afghanistan | Low | Low | Low | - | 1st tertile | 1st tertile | Low | Low | Low | 1st | 3rd tertile | 3rd tertile | - | Low | Low |
| Albania | Low | Middle | Middle | Middle | 3rd tertile | 3rd tertile | Middle | Middle | Middle | 1st | 2nd tertile | 3rd tertile | Low | Low | Middle |
| Algeria | Low | Low | Low | Middle | 2nd tertile | 2nd tertile | Low-middle | Low-middle | Middle |  | 1st tertile | 2nd tertile | Low | Middle | Middle |
| American Samoa | - | High | - | - | - | - | High-middle | Middle | High-middle | 1st | - | - | - | - | - |
| Andorra | High | High | High | High | 2nd tertile | 3rd tertile | High | High | High | 3rd | 3rd tertile | 3rd tertile | - | - | - |
| Angola | Low | Low | Low | Low | 1st tertile | 1st tertile | Low | Low | Low | 1st | 3rd tertile | 3rd tertile | Low | Low | Low |
| Antigua and Barbuda | High | High | High | Middle | 3rd tertile | 2nd tertile | High | High-middle | High-middle | 3rd | 1st tertile | 1st tertile | Medium | Middle | Middle |
| Argentina | Middle | Middle | Middle | High | 3rd tertile | 3rd tertile | High-middle | High-middle | Middle | 1st | 3rd tertile | 3rd tertile | Medium | Middle | Middle |
| Armenia | Middle | Middle | Middle | Low | 1st tertile | 1st tertile | Middle | High-middle | Middle | 3rd | 1st tertile | 2nd tertile | Low | Low | Middle |
| Australia | High | High | High | High | 3rd tertile | 3rd tertile | High | High | High | 3rd | 3rd tertile | 3rd tertile | Medium | High | High |
| Austria | High | High | High | High | 3rd tertile | 3rd tertile | High | High | High |  | 3rd tertile | 3rd tertile | High | High | High |
| Azerbaijan | Low | Low | Low | Low | 1st tertile | 1st tertile | Middle | High-middle | Middle | 3rd | 2nd tertile | 2nd tertile | Low | Middle | Middle |
| Bahamas | High | High | High | High | 3rd tertile | 2nd tertile | High | High-middle | - |  | 2nd tertile | 1st tertile | High | High | High |
| Bahrain | Middle | Middle | Middle | Middle | 2nd tertile | 2nd tertile | High-middle | High-middle | High-middle | 2nd | 1st tertile | 1st tertile | High | High | High |
| Bangladesh | Low | Low | Low | Low | 1st tertile | 1st tertile | Low | Low | Low | 2nd | 2nd tertile | 2nd tertile | Low | Low | Low |
| Barbados | High | High | High | High | 2nd tertile | 2nd tertile | High-middle | High-middle | High-middle |  | 1st tertile | 2nd tertile | Medium | Middle | Middle |
| Belarus | Low | Low | Middle | High | 2nd tertile | 2nd tertile | High-middle | High-middle | High-middle | 3rd | 3rd tertile | 3rd tertile | Low | Middle | Middle |
| Belgium | High | High | High | High | 3rd tertile | 3rd tertile | High | High | High | 1st | 3rd tertile | 3rd tertile | High | High | High |
| Belize | Middle | Middle | Middle | Low | 3rd tertile | 2nd tertile | Middle | Middle | Low-middle | 1st | 1st tertile | 1st tertile | Low | Low | Low |
| Benin | Middle | Middle | Middle | Low | 1st tertile | 1st tertile | Low | Low | Low |  | 1st tertile | 1st tertile | Low | Low | Low |
| Bermuda | High | High | - |  | - | - | High | High | High | 2nd | - | - | High | High | - |
| Bhutan | Middle | Middle | High | Middle | 1st tertile | 1st tertile | Low-middle | Low-middle | Low | 2nd | 1st tertile | 1st tertile | Low | Low | - |
| Bolivia (Plurinational State of) | Middle | Low | Low | Middle | 2nd tertile | 2nd tertile | Low-middle | Low-middle | Low-middle | - | 3rd tertile | 3rd tertile | Low | Low | Low |
| Bosnia and  Herzegovina | Low | Middle | Middle | Middle | 3rd tertile | 3rd tertile | Middle | High-middle | High-middle | 3rd | 2nd tertile | 2nd tertile | Low | Low | Middle |
| Botswana | High | High | High | Middle | 2nd tertile | 3rd tertile | Low-middle | Low-middle | Middle | 1st | 1st tertile | 1st tertile | Low | Middle | Middle |
| Brazil | Middle | Middle | Middle | Middle | 2nd tertile | 2nd tertile | Middle | Middle | Middle |  | 1st tertile | 1st tertile | Low | Middle | Middle |
| Brunei  Darussalam | High | High | High | Low | 1st tertile | 1st tertile | High | High | High | 2nd | - | 1st tertile | High | High | High |
| Bulgaria | Middle | High | High | Middle | 2nd tertile | 2nd tertile | High-middle | High-middle | High-middle | 2nd | 2nd tertile | 2nd tertile | Low | Middle | Middle |
| Burkina Faso | Middle | Middle | Low | Low | 1st tertile | 2nd tertile | Low | Low | Low | 3rd | 2nd tertile | 1st tertile | Low | Low | Low |
| Burundi | Low | Low | Low | Low | 1st tertile | 2nd tertile | Low | Low | Low | 2nd | 3rd tertile | 3rd tertile | Low | Low | Low |
| CÃ´te d'Ivoire | Low | High | High | Middle | 1st tertile | 2nd tertile | Low-middle | Low-middle | Low-middle | 2nd | 1st tertile | 1st tertile | Low | Low | Low |
| Cabo Verde | Low | Low | Low | Low | 1st tertile | 1st tertile | High | Low-middle | - | 1st | 2nd tertile | 2nd tertile | Low | Low | Low |
| Cambodia | High | Low | Low | High | 1st tertile | 1st tertile | Middle | High | High |  | 2nd tertile | 2nd tertile | High | Low | Low |
| Cameroon | - | High | High | - | 3rd tertile | 3rd tertile | Low | Middle | Low-middle | 2nd | 2nd tertile | 3rd tertile | Low | High | High |
| Canada | Low | - | - | Middle | - | - | High-middle | Low | Low | 1st | 3rd tertile | 3rd tertile | Low | Middle | Middle |
| Central  African Republic | High | Low | Low | Middle | 1st tertile | 1st tertile | Low-middle | High-middle | High-middle | 2nd | 1st tertile | 1st tertile | Low | Low | Low |
| Chad | Middle | High | High | Low | 3rd tertile | 3rd tertile | Middle | Middle | Middle | 3rd | 1st tertile | 1st tertile | Low | Middle | Middle |
| Chile | Middle | Middle | Middle | High | 2nd tertile | 2nd tertile | Low | Middle | Middle | 2nd | 2nd tertile | 2nd tertile | Low | Low | Middle |
| China | Low | Middle | Middle | Middle | 3rd tertile | 3rd tertile | Low-middle | Low | Low |  | 3rd tertile | 2nd tertile | Low | Middle | Middle |
| Colombia | Low | Low | Low | Low | 1st tertile | 1st tertile | - | Low-middle | Low-middle | 2nd | 2nd tertile | 2nd tertile | Low | Low | Low |
| Comoros | - | Low | Low | - | 1st tertile | 1st tertile | Middle | - | High-middle |  | 1st tertile | 1st tertile | - | Low | Low |
| Congo | High | Middle | - | High | - | - | Low | Middle | Middle | 3rd | 1st tertile | 1st tertile | Low | - | - |
| Cook Islands | Middle | High | High | High | 3rd tertile | 3rd tertile | High-middle | Low | Low | 3rd | - | - | Medium | Middle | Middle |
| Costa Rica | Middle | High | High | Middle | 3rd tertile | 3rd tertile | High-middle | High-middle | High-middle | 3rd | 3rd tertile | 3rd tertile |  | Middle | High |
| Croatia | High | Low | Middle | Low | 3rd tertile | 3rd tertile | High | High-middle | Middle | 1st | 3rd tertile | 2nd tertile | Medium | - | - |
| Cuba | High | High | High | High | 1st tertile | 1st tertile | High | High | High | 3rd | 3rd tertile | 3rd tertile | Medium | High | High |
| Cyprus | Low | High | High |  | 3rd tertile | 3rd tertile | Low-middle | High | High |  | 2nd tertile | 2nd tertile | Low | High | High |
| Czechia | High | Low | Low | Middle | - | - | Low | Low-middle | Low-middle | 3rd | 3rd tertile | 2nd tertile | Low | Low | Low |
| Democratic People's Republic of Korea | Low | Low | Low | - | - | - | Middle | Low-middle | - | - | 2nd tertile | 2nd tertile | - | - | - |
| Democratic Republic of the Congo | Low | Low | Low | - | - | - | Low | Low | - | 3rd | 1st tertile | 1st tertile | Low | Low | Low |
| Denmark | High | High | High | High | 3rd tertile | 3rd tertile | High | High | High | 1st | 3rd tertile | 3rd tertile | High | High | High |
| Djibouti | Low | Low | Low | Low | 1st tertile | 1st tertile | Low-middle | Low | Low |  | 2nd tertile | 2nd tertile |  | - | Low |
| Dominica | High | High | High | Middle | 2nd tertile | 1st tertile | High-middle | High-middle | High-middle | 3rd | 2nd tertile | 2nd tertile | Low | Low | Middle |
| Dominican Republic | Middle | Middle | Middle | High | 3rd tertile | 3rd tertile | Middle | Middle | Low-middle | 3rd | 2nd tertile | 3rd tertile | Low | Middle | Middle |
| Ecuador | Low | Low | Middle | Low | 2nd tertile | 2nd tertile | Middle | Middle | Middle | 1st | 3rd tertile | 3rd tertile | Low | Low | Middle |
| Egypt | Middle | Low | Low | Low | 1st tertile | 1st tertile | Low-middle | Low-middle | Middle | 2nd | 2nd tertile | 1st tertile | Low | Low | Middle |
| El Salvador | Middle | Middle | Middle | High | 3rd tertile | 3rd tertile | Low-middle | Low-middle | Low-middle | 1st | 2nd tertile | 3rd tertile | Low | Low | Low |
| Equatorial Guinea | Low | Low | Low | Low | 1st tertile | 1st tertile | Low-middle | Low-middle | Middle | 3rd | 1st tertile | 2nd tertile | Low | High | Middle |
| Eritrea | Low | Low | Low | Low | 1st tertile | 1st tertile | Low | Low | Low | 3rd | 3rd tertile | 2nd tertile | Low | Low | - |
| Estonia | High | High | High | Middle | 2nd tertile | 3rd tertile | High-middle | High-middle | High | 1st | 3rd tertile | 3rd tertile | Low | Middle | High |
| Eswatini | - | - | - | Middle | 3rd tertile | 2nd tertile | - | - | Low-middle | 2nd | 2nd tertile | 1st tertile | Low | Low | -Low |
| Ethiopia | Low | Low | Low | Low | 1st tertile | 1st tertile | Low | Low | Low |  | 3rd tertile | 3rd tertile | Low | Low | Low |
| Fiji | Middle | Low | Middle | Middle | 2nd tertile | 1st tertile | Middle | Middle | Middle | 3rd | - | 2nd tertile | Low | Low | Middle |
| Finland | High | High | High | Middle | 3rd tertile | 3rd tertile | High | High | High | 2nd | 3rd tertile | 3rd tertile | High | High | High |
| France | High | High | High | High | 3rd tertile | 3rd tertile | High-middle | High-middle | High | 2nd | 2nd tertile | 3rd tertile | Medium | High | High |
| Gabon | Middle | Low | Low | Low | 1st tertile | 2nd tertile | Low-middle | Low-middle | Middle | 1st | 2nd tertile | 2nd tertile | Medium | Middle | Middle |
| Gambia | Middle | Low | Middle | Low | 2nd tertile | 1st tertile | Low | Low | - | 1st | 1st tertile | 1st tertile | Low | Low | Low |
| Georgia | Low | Middle | High | Low | 1st tertile | 2nd tertile | High-middle | High-middle | Middle | 3rd | 1st tertile | 1st tertile | Low | Low | Middle |
| Germany | High | High | High | High | 3rd tertile | 3rd tertile | High | High | High |  | 3rd tertile | 3rd tertile | High | High | High |
| Ghana | Middle | Middle | Middle | Low | 3rd tertile | 1st tertile | Low-middle | Low-middle | Low-middle | 2nd | 1st tertile | 1st tertile | Low | Low | Low |
| Greece | High | High | High | - | 3rd tertile | 2nd tertile | High-middle | High-middle | Middle |  | 2nd tertile | 2nd tertile | Medium | High | High |
| Greenland | - | High | High | - | - | - | High-middle | Middle | High-middle | 3rd | - | - | - | - | - |
| Grenada | High | High | Middle | Low | 2nd tertile | 2nd tertile | High-middle | High-middle | Middle |  | 2nd tertile | 3rd tertile | Low | Middle | Middle |
| Guam | - | High | - | - | - | - | High | High | High | 2nd | - | - | - | - | - |
| Guatemala | Low | Low | Low | High | 3rd tertile | 3rd tertile | Low-middle | Low-middle | Low-middle | 2nd | 2nd tertile | 2nd tertile | Low | Low | Low |
| Guinea | Low | Low | Low | Low | 1st tertile | 1st tertile | Low | Low | Low | 2nd | - | 2nd tertile | Low | Low | Low |
| Guinea-Bissau | Low | Low | Low | High | 1st tertile | 1st tertile | Low | Low | Low | 3rd | 1st tertile | 1st tertile | Low | Low | Low |
| Guyana | Middle | Middle | Middle | Low | 1st tertile | 2nd tertile | Middle | Middle | Low-middle |  | 3rd tertile | 3rd tertile | Low | Low | Low |
| Haiti | Low | Low | Low | High | 1st tertile | 1st tertile | Low | Low | Low | 2nd | 1st tertile | 1st tertile | Low | Low | Low |
| Honduras | Low | Low | Low | High | 3rd tertile | 2nd tertile | Low-middle | Low-middle | Low | 2nd | 2nd tertile | 2nd tertile | Low | Low | Low |
| Hungary | High | High | High | Middle | 2nd tertile | 2nd tertile | High-middle | High-middle | High-middle | 3rd | 1st tertile | 1st tertile | Medium | Middle | High |
| Iceland | High | High | High | High | 3rd tertile | 3rd tertile | High | High | High | 2nd | 3rd tertile | 3rd tertile | High | High | High |
| India | Middle | Middle | Middle | Low | 1st tertile | 1st tertile | Low-middle | Low-middle | Low-middle | 2nd | 1st tertile | 1st tertile | Low | Low | Low |
| Indonesia | Low | Middle | Middle | Low | 1st tertile | 2nd tertile | Middle | Middle | Middle | 1st | 2nd tertile | 2nd tertile | Low | Low | Middle |
| Iran (Islamic Republic of) | Low | Low | Low | Middle | 3rd tertile | 3rd tertile | Middle | Middle | Middle | 2nd | 1st tertile | 1st tertile | Low | Middle | - |
| Iraq | Low | Low | Low | - | 1st tertile | 1st tertile | Low-middle | Low-middle | Middle | 2nd | 3rd tertile | 2nd tertile | Low | Middle | Middle |
| Ireland | High | High | High | High | 3rd tertile | 3rd tertile | High | High | High | 3rd | 2nd tertile | 2nd tertile | High | High | High |
| Israel | High | High | High | Middle | 2nd tertile | 2nd tertile | High | High-middle | High-middle | 2nd | 2nd tertile | 2nd tertile | Medium | High | High |
| Italy | High | High | High | High | 3rd tertile | 3rd tertile | High | High | High-middle | 3rd | 3rd tertile | 3rd tertile | High | High | High |
| Jamaica | Middle | Middle | Middle | High | 2nd tertile | 3rd tertile | High-middle | Middle | Middle | 1st | 2nd tertile | 2nd tertile | Low | Low | Low |
| Japan | High | High | High | High | 3rd tertile | 3rd tertile | High | High | High | 1st | 2nd tertile | 1st tertile | High | High | High |
| Jordan | Middle | Middle | Middle | High | 3rd tertile | 3rd tertile | Middle | Middle | High-middle | 2nd | 1st tertile | 1st tertile | Low | Low | Low |
| Kazakhstan | Low | Middle | Middle | Middle | 2nd tertile | 1st tertile | High-middle | High-middle | High-middle | 1st | 2nd tertile | 3rd tertile | Low | Middle | High |
| Kenya | Low | Low | Low | Low | 2nd tertile | 2nd tertile | Low-middle | Low-middle | Low-middle | 1st | 1st tertile | 2nd tertile | Low | Low | Low |
| Kiribati | - | Middle | High | Middle | 2nd tertile | 1st tertile | Low-middle | Low-middle | Low-middle | 1st | 1st tertile | 1st tertile | Low | Low | Low |
| Kuwait | Middle | Middle | Middle | Low | 1st tertile | 2nd tertile | High-middle | High | High | 1st | 1st tertile | 1st tertile | High | High | High |
| Kyrgyzstan | Low | Low | Low | Low | 2nd tertile | 1st tertile | Middle | Middle | Low-middle | 3rd | 3rd tertile | 2nd tertile | Low | Low | Low |
| Lao People's Democratic Republic | Low | Low | Low | Low | 1st tertile | 1st tertile | Low-middle | Low-middle | Low | 3rd | 3rd tertile | 3rd tertile | Low | Low | Low |
| Latvia | High | High | High | Middle | 2nd tertile | 2nd tertile | High-middle | High | High | 1st | 2nd tertile | 3rd tertile | Low | Middle | High |
| Lebanon | Middle | Low | Low | Middle | 2nd tertile | 3rd tertile | Middle | High-middle | Middle | 1st | 1st tertile | 1st tertile | Low | Middle | Middle |
| Lesotho | Middle | Middle | Middle | Middle | 2nd tertile | 2nd tertile | Low-middle | Low-middle | Low-middle | 2nd | 3rd tertile | 2nd tertile | Low | Low | Low |
| Liberia | Low | Low | Low | Low | 1st tertile | 1st tertile | Low | Low | Low |  | 2nd tertile | 1st tertile | Low | Low | Low |
| Libya | Low | Low | Low | Low | 1st tertile | - | Middle | Middle | High-middle | 2nd | 1st tertile | 1st tertile | Medium | High | Middle |
| Lithuania | High | High | High | Middle | 2nd tertile | 3rd tertile | High-middle | High-middle | High | 3rd | 2nd tertile | 2nd tertile | Low | Middle | High |
| Luxembourg | High | High | High | High | 3rd tertile | 2nd tertile | High | High | High | 2nd | 2nd tertile | 2nd tertile | High | High | High |
| Madagascar | Middle | Low | Low | Middle | 3rd tertile | 3rd tertile | Low | Low | Low | 2nd | 2nd tertile | 1st tertile | Low | Low | Low |
| Malawi | Middle | Middle | Low | Low | 1st tertile | 2nd tertile | Low | Low | Low | 2nd | 2nd tertile | 2nd tertile | Low | Low | Low |
| Malaysia | Middle | High | High | Low | 1st tertile | 2nd tertile | High-middle | High-middle | High-middle | 1st | 1st tertile | 1st tertile | Medium | Middle | High |
| Maldives | Middle | Middle | Middle | Middle | 3rd tertile | 3rd tertile | Low-middle | Low-middle | Low-middle | 2nd | 1st tertile | 1st tertile | Low | Middle | Middle |
| Mali | Middle | Middle | Low | Low | 1st tertile | 1st tertile | Low | Low | Low | 2nd | 1st tertile | 1st tertile | Low | Low | Low |
| Malta | High | High | High | High | 3rd tertile | 3rd tertile | High-middle | High-middle | High-middle | 1st | 1st tertile | 1st tertile | Medium | High | High |
| Marshall  Islands | - | Middle | Middle | High | 2nd tertile | 2nd tertile | Low-middle | Low-middle | Low-middle | 1st | 1st tertile | 1st tertile | Low | Low | - |
| Mauritania | Middle | Low | Low | Low | 1st tertile | 1st tertile | Low | Low | Low | 1st | 3rd tertile | 2nd tertile | Low | Low | Low |
| Mauritius | High | High | High | Low | 2nd tertile | 2nd tertile | Middle | Middle | Middle | 3rd | 2nd tertile | 2nd tertile | Low | Middle | Middle |
| Mexico | Middle | Middle | Middle | Middle | 2nd tertile | 2nd tertile | Middle | Middle | Middle | 1st | 3rd tertile | 3rd tertile | Medium | Middle | Middle |
| Micronesia (Federated States of) | - | Middle | Middle | Low | 1st tertile | 1st tertile | Low-middle | Low-middle | - | 3rd | 1st tertile | 1st tertile | Low | Low | - |
| Monaco |  |  |  | Low | 2nd tertile | 1st tertile | Middle | Middle | High | 2nd | 3rd tertile | 3rd tertile | - | - | - |
| Mongolia | Middle | Middle | Middle | High | 2nd tertile | 2nd tertile | Middle | Middle | Low-middle | - | 1st tertile | 2nd tertile | Low | Low | Middle |
| Montenegro | - | Middle | Middle | - | - | - | High-middle | High-middle | High-middle | 1st | 1st tertile | 3rd tertile | Low | Middle | Middle |
| Morocco | Middle | Middle | Middle | - | - | - | Low-middle | Low-middle | Low-middle | 3rd | 1st tertile | 2nd tertile | Low | Low | Low |
| Mozambique | Middle | Middle | Low | High | 1st tertile | 1st tertile | Low | Low | Low | - | 3rd tertile | 3rd tertile | Low | Low | Low |
| Myanmar | Low | Low | Low | Low | 1st tertile | 1st tertile | Low | Low-middle | Low-middle | 3rd | 1st tertile | 1st tertile | Low | Low | Low |
| Namibia | Middle | High | Middle | High | 3rd tertile | 2nd tertile | Low-middle | Low-middle | Low-middle | - | 3rd tertile | 3rd tertile | Low | Low | Low |
| Nauru | - | Middle | Middle | Middle | 2nd tertile | 1st tertile | - | - | Low-middle | 1st | 1st tertile | 1st tertile | - | Low | Middle |
| Nepal | Middle | Low | Low | Low | 1st tertile | 1st tertile | Low | Low | Low | 3rd | 3rd tertile | 3rd tertile | Low | Low | Low |
| Netherlands | High | High | High | High | 3rd tertile | 3rd tertile | High | High | High | 3rd | 3rd tertile | 3rd tertile | High | High | High |
| New Zealand | High | High | High | High | 3rd tertile | 3rd tertile | High | High | High | 2nd | 3rd tertile | 3rd tertile | Medium | High | High |
| Nicaragua | Middle | Low | Low | Middle | 3rd tertile | 3rd tertile | Low-middle | Low-middle | Low-middle | 1st | 2nd tertile | 3rd tertile | Low | Low | Low |
| Niger | Low | Low | Low | Middle | 2nd tertile | 2nd tertile | Low | Low | Low | 1st | - | 2nd tertile | Low | Low | Low |
| Nigeria | Low | Low | Low | Low | 1st tertile | 1st tertile | Low | Low-middle | Low-middle | - | 1st tertile | 1st tertile | Low | Low | Low |
| Niue | - | Middle | - | - | - | - | - | - | High-middle | 1st | - | - | - | - | - |
| North Macedonia | Middle | Middle | Middle | High | 3rd tertile | 3rd tertile | High-middle | High-middle | High-middle | - | 3rd tertile | 3rd tertile | Low | Middle | Middle |
| Northern Mariana Islands | - | - | - | - | - | - | High | High | High-middle | 3rd | - | - |  | - | - |
| Norway | High | High | High | High | 3rd tertile | 3rd tertile | High | High | High | - | 3rd tertile | 3rd tertile | High | High | High |
| Oman | Middle | Middle | Middle | Low | 1st tertile | 1st tertile | Middle | Middle | High-middle | - | 1st tertile | 1st tertile | High | High | High |
| Pakistan | Low | Low | Low | Low | 1st tertile | 1st tertile | Low-middle | Low-middle | Low | 1st | 3rd tertile | 2nd tertile | Low | Low | Low |
| Palau | - | Middle | Middle | Middle | 2nd tertile | 3rd tertile | - | - | High-middle | - | 1st tertile | 1st tertile | Low | Middle | - |
| Palestine | - | - | - | - | - | - | Low-middle | Low-middle | Low-middle | 2nd | - | - |  | - | - |
| Panama | Middle | Middle | Middle | High | 3rd tertile | 3rd tertile | High-middle | Middle | Middle | 1st | 1st tertile | 2nd tertile | Low | Middle | High |
| Papua New Guinea | Middle | Low | Low | Middle | 1st tertile | 2nd tertile | Low-middle | Low | Low | 1st | 1st tertile | 1st tertile | Low | Low | Low |
| Paraguay | Low | Low | Middle | Low | 2nd tertile | 2nd tertile | Low-middle | Low-middle | Middle |  | 2nd tertile | 1st tertile | Low | Low | Middle |
| Peru | Middle | Middle | Middle | Middle | 2nd tertile | 3rd tertile | Middle | Middle | Middle | 2nd | 3rd tertile | 3rd tertile | Low | Low | Middle |
| Philippines | Middle | Middle | Middle | Low | 1st tertile | 1st tertile | Middle | Middle | Low-middle | 3rd | 3rd tertile | 3rd tertile | Low | Low | Low |
| Poland | High | High | High | Middle | 2nd tertile | 2nd tertile | High | High | High-middle | 3rd | 2nd tertile | 3rd tertile | Medium | Middle | High |
| Portugal | High | High | High | High | 3rd tertile | 3rd tertile | High-middle | High-middle | High-middle | - | 3rd tertile | 3rd tertile | Medium | High | High |
| Puerto Rico | High | High | Middle | - | - | - | High | High | High | - | - | - | Medium | High | High |
| Qatar | High | High | High | Low | 1st tertile | 1st tertile | High-middle | High-middle | High | 1st | 1st tertile | 1st tertile | High | High | High |
| Republic of  Korea | High | High | High | - | - | - | High | High | - | 2nd | 2nd tertile | 2nd tertile | Medium | High | High |
| Republic of Moldova | Middle | Middle | Middle | - | - | - | Middle | Middle | - | - | 2nd tertile | 2nd tertile | Low | Low | Middle |
| Romania | Middle | Middle | Middle | Middle | 2nd tertile | 3rd tertile | High-middle | High-middle | High-middle | 2nd | 2nd tertile | 2nd tertile | Low | Middle | High |
| Russian Federation | Low | Low | Low | Middle | 2nd tertile | 2nd tertile | High | High | High-middle | 3rd | 2nd tertile | 1st tertile | Low | Middle | High |
| Rwanda | Low | Middle | Middle | Low | 2nd tertile | 2nd tertile | Low | Low | Low | 3rd | 3rd tertile | 3rd tertile | Low | Low | Low |
| Saint Kitts and Nevis | - | High | High | - | - | - | - | - | High-middle | 2nd | 1st tertile | 1st tertile | Medium | Middle | High |
| Saint Lucia | High | High | High | - | - | - | High-middle | High-middle | Middle | 1st | 1st tertile | 2nd tertile | Low | Middle | Middle |
| Saint Vincent and the  Grenadines | High | High | High | - | - | - | High-middle | High-middle | Low-middle | 2nd | 2nd tertile | 1st tertile | Low | Low | Middle |
| Samoa | High | Middle | High | High | 2nd tertile | 2nd tertile | Middle | Middle | Middle | 3rd | 1st tertile | 1st tertile | Low | Low | Low |
| San Marino | - | - | - | High | 3rd tertile | 3rd tertile | - | - | High | 2nd | 2nd tertile | 2nd tertile | High | High | - |
| Sao Tome and Principe | Middle | Middle | Middle | High | 1st tertile | 2nd tertile | Low | Low | Low-middle | - | 2nd tertile | 1st tertile | - | Low | Low |
| Saudi Arabia | Middle | Middle | Middle | Middle | 1st tertile | 2nd tertile | Middle | High-middle | High-middle | 2nd | 1st tertile | 2nd tertile | High | High | High |
| Senegal | Middle | Middle | Middle | Middle | 1st tertile | 1st tertile | Low | Low | Low |  | 3rd tertile | 3rd tertile | Low | Low | Low |
| Serbia | Low | Middle | Middle | High | 3rd tertile | 2nd tertile | High-middle | High-middle | High-middle | 3rd | 3rd tertile | 3rd tertile | Low | Middle | Middle |
| Seychelles | Middle | Middle | High | Low | 2nd tertile | 2nd tertile | High-middle | High-middle | High-middle | 2nd | 3rd tertile | 2nd tertile | Medium | Middle | High |
| Sierra Leone | Low | Low | Low | High | 1st tertile | 2nd tertile | Low | Low | Low | 1st | 2nd tertile | 1st tertile | Low | Low | Low |
| Singapore | High | High | High | Low | 2nd tertile | 3rd tertile | High | High | High | 3rd | 3rd tertile | 2nd tertile | High | High | High |
| Slovakia | High | High | High | Middle | 3rd tertile | 3rd tertile | High-middle | High | High | - | 2nd tertile | 2nd tertile | Medium | Middle | High |
| Slovenia | High | High | High | High | 3rd tertile | 3rd tertile | High | High | High | 1st | 2nd tertile | 3rd tertile | Medium | High | High |
| Solomon Islands | Low | Middle | Middle | High | 2nd tertile | 1st tertile | Low-middle | Low | Low | - | 1st tertile | 1st tertile | Low | Low | Low |
| Somalia | Low | Low | Low | - | - | - | Low | Low | Low | 3rd | 1st tertile | 2nd tertile | - | - | - |
| South Africa | High | High | Middle | Middle | 3rd tertile | 3rd tertile | Middle | Middle | Middle | - | 3rd tertile | 3rd tertile | Low | Middle | Middle |
| South Sudan | - | - | Low | - | - | 1st tertile | Low | Low | Low | 3rd | - | 3rd tertile | Low | Low | Low |
| Spain | High | High | High | High | 3rd tertile | 3rd tertile | High-middle | High-middle | High-middle | - | 3rd tertile | 3rd tertile | Medium | High | High |
| Sri Lanka | Middle | Middle | Middle | Middle | 2nd tertile | 2nd tertile | Middle | Middle | Middle | - | 1st tertile | 1st tertile | Low | Low | Middle |
| Sudan | Low | Low | Low | High | 2nd tertile | 2nd tertile | Low | Low | Low-middle | - | 3rd tertile | - | Low | Low | Low |
| Suriname | Middle | Middle | Middle | Middle | 2nd tertile | 2nd tertile | Middle | Middle | Middle | 3rd | 1st tertile | 3rd tertile | Low | Middle | Middle |
| Sweden | High | High | High | Middle | 3rd tertile | 3rd tertile | High | High | High | 3rd | 3rd tertile | 3rd tertile | High | High | High |
| Switzerland | High | High | High | Middle | 2nd tertile | 2nd tertile | High | High | High | 2nd | 3rd tertile | 3rd tertile | High | High | High |
| Syrian Arab Republic | Low | Low | Low | Low | 1st tertile | - | Low-middle | Low-middle | Low-middle | - | 2nd tertile | 1st tertile | - | - | - |
| Taiwan (Province of China) | High | High | High | - | - | - | High | High | High | 3rd | - | - | - | - | - |
| Tajikistan | Low | Low | Low | Low | 1st tertile | 1st tertile | Low-middle | Low-middle | Low-middle | 1st | 2nd tertile | 2nd tertile | Low | Low | Low |
| Thailand | High | Middle | Middle | High | 3rd tertile | 3rd tertile | Middle | Middle | Middle | - | 2nd tertile | 1st tertile | Low | Middle | Middle |
| Timor-Leste | - | Low | Middle |  | 1st tertile | 1st tertile | Low | Low | Low-middle | 1st | 3rd tertile | 3rd tertile | Low | Low | Low |
| Togo | Low | Low | Low | Low | 2nd tertile | 1st tertile | Low | Low | Low | 1st | 1st tertile | 1st tertile | Low | Low | Low |
| Tokelau | - | - | - | - | - | - | - | - | Low-middle | - | - | - | - | - | - |
| Tonga | - | Middle | Middle | - | - | - | Low-middle | Low-middle | Middle | 2nd | 1st tertile | 1st tertile | Low | Low | - |
| Trinidad and  Tobago | High | Middle | Middle | Low | 2nd tertile | 2nd tertile | High-middle | High-middle | High-middle | 1st | 3rd tertile | 3rd tertile | Medium | High | High |
| Tunisia | Middle | Middle | Middle | Middle | 3rd tertile | 3rd tertile | Middle | Middle | Middle | - | 3rd tertile | 2nd tertile | Low | Low | Middle |
| Turkey | Middle | Middle | Middle | Middle | 2nd tertile | 2nd tertile | Middle | Middle | High-middle | 3rd | 1st tertile | 2nd tertile | Low | Middle | High |
| Turkmenistan | Low | Low | Low | High | 2nd tertile | 2nd tertile | Middle | High-middle | Middle | 1st | 2nd tertile | 2nd tertile | Low | Low | - |
| Tuvalu | - | Middle | High | High | 3rd tertile | 2nd tertile |  | - | Low-middle | 3rd | 1st tertile | 1st tertile | Low | Low | Low |
| Uganda | Low | Low | Low | Middle | 2nd tertile | 1st tertile | Low | Low | Low | 2nd | 3rd tertile | 3rd tertile | Low | Low | Low |
| Ukraine | Low | Low | Low | Low | 2nd tertile | 1st tertile | High-middle | High-middle | High-middle | 1st | 1st tertile | 2nd tertile | Low | Low | Middle |
| United Arab Emirates | High | High | High | Middle | 2nd tertile | 1st tertile | High | High | High | 3rd | 3rd tertile | 3rd tertile | High | High | High |
| United Kingdom | High | High | High | High | 3rd tertile | 3rd tertile | High | High | High | - | 3rd tertile | 3rd tertile | High | High | High |
| United Republic of Tanzania | Middle | Middle | Low | - | - | - | Low | Low | - | - | - | 3rd tertile | Low | Low | Low |
| United States of America | High | High | - | High | - | - | High | High | - | - | 2nd tertile | 2nd tertile | High | - | - |
| United States Virgin Islands | - | High | High | - | 3rd tertile | 3rd tertile | High | High | High | 2nd | - | - |  | High | High |
| Uruguay | High | High | High | High | 3rd tertile | 3rd tertile | High-middle | Middle | Middle | 1st | 2nd tertile | 2nd tertile | Low | Middle | Middle |
| Uzbekistan | Low | Low | Low | Low | 2nd tertile | 2nd tertile | Middle | Middle | Low-middle | 1st | 3rd tertile | 1st tertile | Low | Low | Low |
| Vanuatu | Middle | Middle | Middle | Middle | 1st tertile | 1st tertile | Low-middle | Low-middle | Low | - | 1st tertile | 1st tertile | Low | Low | Low |
| Venezuela (Bolivarian Republic of) | Low | Low | Low | High | 2nd tertile | 1st tertile | High-middle | Middle | Low-middle | 3rd | 2nd tertile | 2nd tertile | Medium | Middle | - |
| Viet Nam | Middle | Low | Middle | Middle | 2nd tertile | 2nd tertile | Low-middle | Low-middle | Low-middle | 1st | 3rd tertile | 2nd tertile | Low | Low | Low |
| Yemen | Low | Low | Low | Middle | 1st tertile | - | Low | Low | Low | 2nd | 1st tertile | 1st tertile | Low | Low | - |
| Zambia | Middle | Middle | Middle | High | 1st tertile | 1st tertile | Low | Low | Low-middle | 2nd | 2nd tertile | 2nd tertile | Low | Low | Low |
| Zimbabwe | Low | Low | Low | - | 3rd tertile | 3rd tertile | Low-middle | Low-middle | Low | - | 2nd tertile | 3rd tertile | Low | Low | Low |

|  |  |  |  |  |  |  |  |  |  |  |
| --- | --- | --- | --- | --- | --- | --- | --- | --- | --- | --- |
|  |  |  |  |  |  |  |  |  |  |  |
|  |  |  |  |  |  |  |  |  |  |  |
|  |  |  |  |  |  |  |  |  |  |  |
|  |  |  |  |  |  |  |  |  |  |  |
|  |  |  |  |  |  |  |  |  |  |  |
|  |  |  |  |  |  |  |  |  |  |  |
|  |  |  |  |  |  |  |  |  |  |  |
|  |  |  |  |  |  |  |  |  |  |  |
|  |  |  |  |  |  |  |  |  |  |  |
|  |  |  |  |  |  |  |  |  |  |  |
|  |  |  |  |  |  |  |  |  |  |  |
|  |  |  |  |  |  |  |  |  |  |  |
|  |  |  |  |  |  |  |  |  |  |  |
|  |  |  |  |  |  |  |  |  |  |  |
|  |  |  |  |  |  |  |  |  |  |  |
|  |  |  |  |  |  |  |  |  |  |  |
|  |  |  |  |  |  |  |  |  |  |  |
|  |  |  |  |  |  |  |  |  |  |  |
|  |  |  |  |  |  |  |  |  |  |  |
|  |  |  |  |  |  |  |  |  |  |  |
|  |  |  |  |  |  |  |  |  |  |  |
|  |  |  |  |  |  |  |  |  |  |  |
|  |  |  |  |  |  |  |  |  |  |  |
|  |  |  |  |  |  |  |  |  |  |  |
|  |  |  |  |  |  |  |  |  |  |  |
|  |  |  |  |  |  |  |  |  |  |  |
|  |  |  |  |  |  |  |  |  |  |  |
|  |  |  |  |  |  |  |  |  |  |  |
|  |  |  |  |  |  |  |  |  |  |  |
|  |  |  |  |  |  |  |  |  |  |  |
|  |  |  |  |  |  |  |  |  |  |  |
|  |  |  |  |  |  |  |  |  |  |  |
|  |  |  |  |  |  |  |  |  |  |  |
|  |  |  |  |  |  |  |  |  |  |  |
|  |  |  |  |  |  |  |  |  |  |  |
|  |  |  |  |  |  |  |  |  |  |  |
|  |  |  |  |  |  |  |  |  |  |  |
|  |  |  |  |  |  |  |  |  |  |  |
|  |  |  |  |  |  |  |  |  |  |  |
|  |  |  |  |  |  |  |  |  |  |  |
|  |  |  |  |  |  |  |  |  |  |  |
|  |  |  |  |  |  |  |  |  |  |  |
|  |  |  |  |  |  |  |  |  |  |  |
|  |  |  |  |  |  |  |  |  |  |  |
|  |  |  |  |  |  |  |  |  |  |  |
|  |  |  |  |  |  |  |  |  |  |  |
|  |  |  |  |  |  |  |  |  |  |  |
|  |  |  |  |  |  |  |  |  |  |  |
|  |  |  |  |  |  |  |  |  |  |  |
|  |  |  |  |  |  |  |  |  |  |  |
|  |  |  |  |  |  |  |  |  |  |  |
|  |  |  |  |  |  |  |  |  |  |  |
|  |  |  |  |  |  |  |  |  |  |  |
|  |  |  |  |  |  |  |  |  |  |  |
|  |  |  |  |  |  |  |  |  |  |  |
|  |  |  |  |  |  |  |  |  |  |  |
|  |  |  |  |  |  |  |  |  |  |  |
|  |  |  |  |  |  |  |  |  |  |  |
|  |  |  |  |  |  |  |  |  |  |  |
|  |  |  |  |  |  |  |  |  |  |  |
|  |  |  |  |  |  |  |  |  |  |  |
|  |  |  |  |  |  |  |  |  |  |  |
|  |  |  |  |  |  |  |  |  |  |  |
|  |  |  |  |  |  |  |  |  |  |  |
|  |  |  |  |  |  |  |  |  |  |  |
|  |  |  |  |  |  |  |  |  |  |  |
|  |  |  |  |  |  |  |  |  |  |  |
|  |  |  |  |  |  |  |  |  |  |  |
|  |  |  |  |  |  |  |  |  |  |  |
|  |  |  |  |  |  |  |  |  |  |  |
|  |  |  |  |  |  |  |  |  |  |  |
|  |  |  |  |  |  |  |  |  |  |  |
|  |  |  |  |  |  |  |  |  |  |  |
|  |  |  |  |  |  |  |  |  |  |  |
|  |  |  |  |  |  |  |  |  |  |  |
|  |  |  |  |  |  |  |  |  |  |  |
|  |  |  |  |  |  |  |  |  |  |  |
|  |  |  |  |  |  |  |  |  |  |  |
|  |  |  |  |  |  |  |  |  |  |  |
|  |  |  |  |  |  |  |  |  |  |  |
|  |  |  |  |  |  |  |  |  |  |  |
|  |  |  |  |  |  |  |  |  |  |  |
|  |  |  |  |  |  |  |  |  |  |  |
|  |  |  |  |  |  |  |  |  |  |  |
|  |  |  |  |  |  |  |  |  |  |  |
|  |  |  |  |  |  |  |  |  |  |  |
|  |  |  |  |  |  |  |  |  |  |  |
|  |  |  |  |  |  |  |  |  |  |  |
|  |  |  |  |  |  |  |  |  |  |  |
|  |  |  |  |  |  |  |  |  |  |  |
|  |  |  |  |  |  |  |  |  |  |  |
|  |  |  |  |  |  |  |  |  |  |  |
|  |  |  |  |  |  |  |  |  |  |  |
|  |  |  |  |  |  |  |  |  |  |  |
|  |  |  |  |  |  |  |  |  |  |  |
|  |  |  |  |  |  |  |  |  |  |  |
|  |  |  |  |  |  |  |  |  |  |  |
|  |  |  |  |  |  |  |  |  |  |  |
|  |  |  |  |  |  |  |  |  |  |  |
|  |  |  |  |  |  |  |  |  |  |  |
|  |  |  |  |  |  |  |  |  |  |  |
|  |  |  |  |  |  |  |  |  |  |  |
|  |  |  |  |  |  |  |  |  |  |  |
|  |  |  |  |  |  |  |  |  |  |  |
|  |  |  |  |  |  |  |  |  |  |  |
|  |  |  |  |  |  |  |  |  |  |  |
|  |  |  |  |  |  |  |  |  |  |  |
|  |  |  |  |  |  |  |  |  |  |  |
|  |  |  |  |  |  |  |  |  |  |  |
|  |  |  |  |  |  |  |  |  |  |  |
|  |  |  |  |  |  |  |  |  |  |  |
|  |  |  |  |  |  |  |  |  |  |  |
|  |  |  |  |  |  |  |  |  |  |  |
|  |  |  |  |  |  |  |  |  |  |  |
|  |  |  |  |  |  |  |  |  |  |  |
|  |  |  |  |  |  |  |  |  |  |  |
|  |  |  |  |  |  |  |  |  |  |  |
|  |  |  |  |  |  |  |  |  |  |  |
|  |  |  |  |  |  |  |  |  |  |  |
|  |  |  |  |  |  |  |  |  |  |  |
|  |  |  |  |  |  |  |  |  |  |  |
|  |  |  |  |  |  |  |  |  |  |  |
|  |  |  |  |  |  |  |  |  |  |  |
|  |  |  |  |  |  |  |  |  |  |  |
|  |  |  |  |  |  |  |  |  |  |  |
|  |  |  |  |  |  |  |  |  |  |  |
|  |  |  |  |  |  |  |  |  |  |  |
|  |  |  |  |  |  |  |  |  |  |  |
|  |  |  |  |  |  |  |  |  |  |  |
|  |  |  |  |  |  |  |  |  |  |  |
|  |  |  |  |  |  |  |  |  |  |  |
|  |  |  |  |  |  |  |  |  |  |  |
|  |  |  |  |  |  |  |  |  |  |  |
|  |  |  |  |  |  |  |  |  |  |  |
|  |  |  |  |  |  |  |  |  |  |  |
|  |  |  |  |  |  |  |  |  |  |  |
|  |  |  |  |  |  |  |  |  |  |  |
|  |  |  |  |  |  |  |  |  |  |  |
|  |  |  |  |  |  |  |  |  |  |  |
|  |  |  |  |  |  |  |  |  |  |  |
|  |  |  |  |  |  |  |  |  |  |  |
|  |  |  |  |  |  |  |  |  |  |  |
|  |  |  |  |  |  |  |  |  |  |  |
|  |  |  |  |  |  |  |  |  |  |  |
|  |  |  |  |  |  |  |  |  |  |  |
|  |  |  |  |  |  |  |  |  |  |  |
|  |  |  |  |  |  |  |  |  |  |  |
|  |  |  |  |  |  |  |  |  |  |  |
|  |  |  |  |  |  |  |  |  |  |  |
|  |  |  |  |  |  |  |  |  |  |  |
|  |  |  |  |  |  |  |  |  |  |  |
|  |  |  |  |  |  |  |  |  |  |  |
|  |  |  |  |  |  |  |  |  |  |  |
|  |  |  |  |  |  |  |  |  |  |  |
|  |  |  |  |  |  |  |  |  |  |  |
|  |  |  |  |  |  |  |  |  |  |  |
|  |  |  |  |  |  |  |  |  |  |  |
|  |  |  |  |  |  |  |  |  |  |  |
|  |  |  |  |  |  |  |  |  |  |  |
|  |  |  |  |  |  |  |  |  |  |  |
|  |  |  |  |  |  |  |  |  |  |  |
|  |  |  |  |  |  |  |  |  |  |  |
|  |  |  |  |  |  |  |  |  |  |  |
|  |  |  |  |  |  |  |  |  |  |  |
|  |  |  |  |  |  |  |  |  |  |  |
|  |  |  |  |  |  |  |  |  |  |  |
|  |  |  |  |  |  |  |  |  |  |  |
|  |  |  |  |  |  |  |  |  |  |  |
|  |  |  |  |  |  |  |  |  |  |  |
|  |  |  |  |  |  |  |  |  |  |  |
|  |  |  |  |  |  |  |  |  |  |  |
|  |  |  |  |  |  |  |  |  |  |  |
|  |  |  |  |  |  |  |  |  |  |  |
|  |  |  |  |  |  |  |  |  |  |  |
|  |  |  |  |  |  |  |  |  |  |  |
|  |  |  |  |  |  |  |  |  |  |  |
|  |  |  |  |  |  |  |  |  |  |  |
|  |  |  |  |  |  |  |  |  |  |  |
|  |  |  |  |  |  |  |  |  |  |  |
|  |  |  |  |  |  |  |  |  |  |  |
|  |  |  |  |  |  |  |  |  |  |  |
|  |  |  |  |  |  |  |  |  |  |  |
|  |  |  |  |  |  |  |  |  |  |  |
|  |  |  |  |  |  |  |  |  |  |  |
|  |  |  |  |  |  |  |  |  |  |  |
|  |  |  |  |  |  |  |  |  |  |  |
|  |  |  |  |  |  |  |  |  |  |  |
|  |  |  |  |  |  |  |  |  |  |  |
|  |  |  |  |  |  |  |  |  |  |  |
|  |  |  |  |  |  |  |  |  |  |  |
|  |  |  |  |  |  |  |  |  |  |  |
|  |  |  |  |  |  |  |  |  |  |  |
|  |  |  |  |  |  |  |  |  |  |  |
|  |  |  |  |  |  |  |  |  |  |  |
|  |  |  |  |  |  |  |  |  |  |  |
|  |  |  |  |  |  |  |  |  |  |  |
|  |  |  |  |  |  |  |  |  |  |  |
|  |  |  |  |  |  |  |  |  |  |  |
|  |  |  |  |  |  |  |  |  |  |  |
|  |  |  |  |  |  |  |  |  |  |  |
|  |  |  |  |  |  |  |  |  |  |  |
|  |  |  |  |  |  |  |  |  |  |  |
|  |  |  |  |  |  |  |  |  |  |  |
|  |  |  |  |  |  |  |  |  |  |  |
|  |  |  |  |  |  |  |  |  |  |  |
|  |  |  |  |  |  |  |  |  |  |  |
|  |  |  |  |  |  |  |  |  |  |  |
|  |  |  |  |  |  |  |  |  |  |  |
|  |  |  |  |  |  |  |  |  |  |  |
|  |  |  |  |  |  |  |  |  |  |  |
|  |  |  |  |  |  |  |  |  |  |  |
|  |  |  |  |  |  |  |  |  |  |  |
|  |  |  |  |  |  |  |  |  |  |  |
|  |  |  |  |  |  |  |  |  |  |  |
|  |  |  |  |  |  |  |  |  |  |  |
|  |  |  |  |  |  |  |  |  |  |  |
|  |  |  |  |  |  |  |  |  |  |  |
|  |  |  |  |  |  |  |  |  |  |  |
|  |  |  |  |  |  |  |  |  |  |  |
|  |  |  |  |  |  |  |  |  |  |  |
|  |  |  |  |  |  |  |  |  |  |  |
|  |  |  |  |  |  |  |  |  |  |  |
|  |  |  |  |  |  |  |  |  |  |  |
|  |  |  |  |  |  |  |  |  |  |  |
|  |  |  |  |  |  |  |  |  |  |  |
|  |  |  |  |  |  |  |  |  |  |  |
|  |  |  |  |  |  |  |  |  |  |  |
|  |  |  |  |  |  |  |  |  |  |  |
|  |  |  |  |  |  |  |  |  |  |  |
|  |  |  |  |  |  |  |  |  |  |  |
|  |  |  |  |  |  |  |  |  |  |  |
|  |  |  |  |  |  |  |  |  |  |  |
|  |  |  |  |  |  |  |  |  |  |  |
|  |  |  |  |  |  |  |  |  |  |  |
|  |  |  |  |  |  |  |  |  |  |  |
|  |  |  |  |  |  |  |  |  |  |  |
|  |  |  |  |  |  |  |  |  |  |  |
|  |  |  |  |  |  |  |  |  |  |  |
|  |  |  |  |  |  |  |  |  |  |  |
|  |  |  |  |  |  |  |  |  |  |  |
|  |  |  |  |  |  |  |  |  |  |  |
|  |  |  |  |  |  |  |  |  |  |  |
|  |  |  |  |  |  |  |  |  |  |  |
|  |  |  |  |  |  |  |  |  |  |  |
|  |  |  |  |  |  |  |  |  |  |  |
|  |  |  |  |  |  |  |  |  |  |  |
|  |  |  |  |  |  |  |  |  |  |  |
|  |  |  |  |  |  |  |  |  |  |  |
|  |  |  |  |  |  |  |  |  |  |  |
|  |  |  |  |  |  |  |  |  |  |  |
|  |  |  |  |  |  |  |  |  |  |  |
|  |  |  |  |  |  |  |  |  |  |  |
|  |  |  |  |  |  |  |  |  |  |  |
|  |  |  |  |  |  |  |  |  |  |  |
|  |  |  |  |  |  |  |  |  |  |  |
|  |  |  |  |  |  |  |  |  |  |  |
|  |  |  |  |  |  |  |  |  |  |  |
|  |  |  |  |  |  |  |  |  |  |  |
|  |  |  |  |  |  |  |  |  |  |  |
|  |  |  |  |  |  |  |  |  |  |  |
|  |  |  |  |  |  |  |  |  |  |  |
|  |  |  |  |  |  |  |  |  |  |  |
|  |  |  |  |  |  |  |  |  |  |  |
|  |  |  |  |  |  |  |  |  |  |  |
|  |  |  |  |  |  |  |  |  |  |  |
|  |  |  |  |  |  |  |  |  |  |  |
|  |  |  |  |  |  |  |  |  |  |  |
|  |  |  |  |  |  |  |  |  |  |  |
|  |  |  |  |  |  |  |  |  |  |  |
|  |  |  |  |  |  |  |  |  |  |  |
|  |  |  |  |  |  |  |  |  |  |  |
|  |  |  |  |  |  |  |  |  |  |  |
|  |  |  |  |  |  |  |  |  |  |  |
|  |  |  |  |  |  |  |  |  |  |  |
|  |  |  |  |  |  |  |  |  |  |  |
|  |  |  |  |  |  |  |  |  |  |  |
|  |  |  |  |  |  |  |  |  |  |  |
|  |  |  |  |  |  |  |  |  |  |  |
|  |  |  |  |  |  |  |  |  |  |  |
|  |  |  |  |  |  |  |  |  |  |  |
|  |  |  |  |  |  |  |  |  |  |  |
|  |  |  |  |  |  |  |  |  |  |  |
|  |  |  |  |  |  |  |  |  |  |  |
|  |  |  |  |  |  |  |  |  |  |  |
|  |  |  |  |  |  |  |  |  |  |  |
|  |  |  |  |  |  |  |  |  |  |  |
|  |  |  |  |  |  |  |  |  |  |  |
|  |  |  |  |  |  |  |  |  |  |  |
|  |  |  |  |  |  |  |  |  |  |  |
|  |  |  |  |  |  |  |  |  |  |  |
|  |  |  |  |  |  |  |  |  |  |  |
|  |  |  |  |  |  |  |  |  |  |  |
|  |  |  |  |  |  |  |  |  |  |  |
|  |  |  |  |  |  |  |  |  |  |  |
|  |  |  |  |  |  |  |  |  |  |  |
|  |  |  |  |  |  |  |  |  |  |  |
|  |  |  |  |  |  |  |  |  |  |  |
|  |  |  |  |  |  |  |  |  |  |  |
|  |  |  |  |  |  |  |  |  |  |  |
|  |  |  |  |  |  |  |  |  |  |  |
|  |  |  |  |  |  |  |  |  |  |  |
|  |  |  |  |  |  |  |  |  |  |  |
|  |  |  |  |  |  |  |  |  |  |  |
|  |  |  |  |  |  |  |  |  |  |  |
|  |  |  |  |  |  |  |  |  |  |  |
|  |  |  |  |  |  |  |  |  |  |  |
|  |  |  |  |  |  |  |  |  |  |  |
|  |  |  |  |  |  |  |  |  |  |  |
|  |  |  |  |  |  |  |  |  |  |  |
|  |  |  |  |  |  |  |  |  |  |  |
|  |  |  |  |  |  |  |  |  |  |  |
|  |  |  |  |  |  |  |  |  |  |  |
|  |  |  |  |  |  |  |  |  |  |  |
|  |  |  |  |  |  |  |  |  |  |  |
|  |  |  |  |  |  |  |  |  |  |  |
|  |  |  |  |  |  |  |  |  |  |  |
|  |  |  |  |  |  |  |  |  |  |  |
|  |  |  |  |  |  |  |  |  |  |  |
|  |  |  |  |  |  |  |  |  |  |  |
|  |  |  |  |  |  |  |  |  |  |  |
|  |  |  |  |  |  |  |  |  |  |  |
|  |  |  |  |  |  |  |  |  |  |  |
|  |  |  |  |  |  |  |  |  |  |  |
|  |  |  |  |  |  |  |  |  |  |  |
|  |  |  |  |  |  |  |  |  |  |  |
|  |  |  |  |  |  |  |  |  |  |  |
|  |  |  |  |  |  |  |  |  |  |  |
|  |  |  |  |  |  |  |  |  |  |  |
|  |  |  |  |  |  |  |  |  |  |  |
|  |  |  |  |  |  |  |  |  |  |  |
|  |  |  |  |  |  |  |  |  |  |  |
|  |  |  |  |  |  |  |  |  |  |  |
|  |  |  |  |  |  |  |  |  |  |  |
|  |  |  |  |  |  |  |  |  |  |  |
|  |  |  |  |  |  |  |  |  |  |  |
|  |  |  |  |  |  |  |  |  |  |  |
|  |  |  |  |  |  |  |  |  |  |  |
|  |  |  |  |  |  |  |  |  |  |  |
|  |  |  |  |  |  |  |  |  |  |  |
|  |  |  |  |  |  |  |  |  |  |  |
|  |  |  |  |  |  |  |  |  |  |  |
|  |  |  |  |  |  |  |  |  |  |  |
|  |  |  |  |  |  |  |  |  |  |  |
|  |  |  |  |  |  |  |  |  |  |  |
|  |  |  |  |  |  |  |  |  |  |  |
|  |  |  |  |  |  |  |  |  |  |  |
|  |  |  |  |  |  |  |  |  |  |  |
|  |  |  |  |  |  |  |  |  |  |  |
|  |  |  |  |  |  |  |  |  |  |  |
|  |  |  |  |  |  |  |  |  |  |  |
|  |  |  |  |  |  |  |  |  |  |  |
|  |  |  |  |  |  |  |  |  |  |  |
|  |  |  |  |  |  |  |  |  |  |  |
|  |  |  |  |  |  |  |  |  |  |  |
|  |  |  |  |  |  |  |  |  |  |  |
|  |  |  |  |  |  |  |  |  |  |  |
|  |  |  |  |  |  |  |  |  |  |  |
|  |  |  |  |  |  |  |  |  |  |  |
|  |  |  |  |  |  |  |  |  |  |  |
|  |  |  |  |  |  |  |  |  |  |  |
|  |  |  |  |  |  |  |  |  |  |  |
|  |  |  |  |  |  |  |  |  |  |  |
|  |  |  |  |  |  |  |  |  |  |  |
|  |  |  |  |  |  |  |  |  |  |  |
|  |  |  |  |  |  |  |  |  |  |  |
|  |  |  |  |  |  |  |  |  |  |  |
|  |  |  |  |  |  |  |  |  |  |  |
|  |  |  |  |  |  |  |  |  |  |  |
|  |  |  |  |  |  |  |  |  |  |  |
|  |  |  |  |  |  |  |  |  |  |  |
|  |  |  |  |  |  |  |  |  |  |  |
|  |  |  |  |  |  |  |  |  |  |  |
|  |  |  |  |  |  |  |  |  |  |  |
|  |  |  |  |  |  |  |  |  |  |  |
|  |  |  |  |  |  |  |  |  |  |  |
|  |  |  |  |  |  |  |  |  |  |  |
|  |  |  |  |  |  |  |  |  |  |  |
|  |  |  |  |  |  |  |  |  |  |  |
|  |  |  |  |  |  |  |  |  |  |  |
|  |  |  |  |  |  |  |  |  |  |  |
|  |  |  |  |  |  |  |  |  |  |  |
|  |  |  |  |  |  |  |  |  |  |  |
|  |  |  |  |  |  |  |  |  |  |  |
|  |  |  |  |  |  |  |  |  |  |  |
|  |  |  |  |  |  |  |  |  |  |  |
|  |  |  |  |  |  |  |  |  |  |  |
|  |  |  |  |  |  |  |  |  |  |  |
|  |  |  |  |  |  |  |  |  |  |  |
|  |  |  |  |  |  |  |  |  |  |  |
|  |  |  |  |  |  |  |  |  |  |  |
|  |  |  |  |  |  |  |  |  |  |  |
|  |  |  |  |  |  |  |  |  |  |  |
|  |  |  |  |  |  |  |  |  |  |  |
|  |  |  |  |  |  |  |  |  |  |  |
|  |  |  |  |  |  |  |  |  |  |  |
|  |  |  |  |  |  |  |  |  |  |  |
|  |  |  |  |  |  |  |  |  |  |  |
|  |  |  |  |  |  |  |  |  |  |  |

Sources: World Bank and GHDX (SDI).

SDI = sociodemographic index.

GDP = gross domestic product per capital.
