# Supplementary material for: Structural determinants of inequalities in untreated dental caries in the Global Burden of Disease Study
Source: PLoS One. 2025 Jun 3;20(6):e0325138. doi: 10.1371/journal.pone.0325138 (PMC12132969; doi:10.1371/journal.pone.0325138)
Supplement: S2 Appendix B — (DOCX) [file pone.0325138.s002.docx]

Appendix B – Prevalence, incidence and YLDs of caries in the primary dentition in 2000, 2010 and 2019 by country.

|  | **Deciduous caries <5 years / 2000** | | | **Deciduous caries <5 years / 2010** | | | **Deciduous caries <5 years / 2019** | | |
| --- | --- | --- | --- | --- | --- | --- | --- | --- | --- |
| **Countries** | Incidence | Prevalence | YLDs | Incidence | Prevalence | YLDs | Incidence | Prevalence | YLDs |
| **Afghanistan** | 41765.04 | 31276.12 | 12.005 | 49198.07 | 38679.34 | 1.478.773 | 49048.09 | 38633.03 | 1.477.548 |
| **Albania** | 50620 | 43431.69 | 1.661.072 | 50077.98 | 42770.77 | 1.632.131 | 50119.05 | 43082.88 | 1.646.464 |
| **Algeria** | 41427.05 | 31236.7 | 119.342 | 48080.71 | 36815.44 | 1.407.119 | 49435.88 | 37516.97 | 1.436.424 |
| **American Samoa** | 46896 | 38900.13 | 1.494.451 | 48423.44 | 40629.51 | 1.558.833 | 48319.96 | 40115.46 | 153.554 |
| **Andorra** | 31398.14 | 17012.54 | 6.511.227 | 40274.4 | 20260.38 | 7.761.172 | 39981.69 | 19972.5 | 7.676.268 |
| **Angola** | 41400.34 | 31017.8 | 1.182.353 | 43864.02 | 32676.07 | 1.244.884 | 44363.15 | 33197.75 | 1.266.259 |
| **Antigua and Barbuda** | 46841.79 | 36812.11 | 1.404.926 | 53223.1 | 42138.16 | 1.612.013 | 53264.43 | 42188.45 | 1.615.192 |
| **Argentina** | 41030.55 | 30780.35 | 1.179.888 | 46968.15 | 31949.29 | 1.221.948 | 48763.18 | 35355.27 | 1.356.531 |
| **Armenia** | 49864.87 | 43762.46 | 1.670.262 | 50699.16 | 42479.78 | 1.618.363 | 52222.97 | 44039.44 | 1.673.692 |
| **Australia** | 11476.37 | 12116.92 | 4.635.462 | 32527.99 | 25517.01 | 9.839.928 | 43641.97 | 34058.71 | 1.305.485 |
| **Austria** | 31638.35 | 17347.77 | 6.664.447 | 38249.97 | 19424.59 | 74.579 | 38942.04 | 19592.56 | 7.509.808 |
| **Azerbaijan** | 50437.92 | 44329.38 | 1.690.725 | 50565.42 | 42316.39 | 1.619.624 | 52521.22 | 43634.55 | 1.675.546 |
| **Bahamas** | 46758.49 | 36187.55 | 1.378.282 | 54035.59 | 42691.8 | 1.635.044 | 53780.18 | 42293.06 | 1.619.611 |
| **Bahrain** | 41223.44 | 30295.44 | 115.987 | 49300.09 | 36106.26 | 1.384.741 | 50328.17 | 37266.56 | 142.784 |
| **Bangladesh** | 42781.27 | 38713.88 | 14.738 | 47073.68 | 39232.45 | 1.498.897 | 47207.56 | 38779.72 | 1.483.022 |
| **Barbados** | 45651.51 | 36031.19 | 1.383.546 | 53263.99 | 42565.59 | 1.627.905 | 53307.87 | 42299.56 | 1.623.341 |
| **Belarus** | 47532.77 | 42509.56 | 1.622.364 | 49994.68 | 42511.81 | 1.634.386 | 51449.8 | 43897.45 | 1.688.705 |
| **Belgium** | 13455.65 | 8.067.661 | 3.089.873 | 39071.59 | 20380.97 | 7.827.973 | 39642.63 | 20586.34 | 789.993 |
| **Belize** | 46072.04 | 36517.93 | 1.400.201 | 53299.37 | 42511.71 | 1.624.806 | 52872.14 | 42354.91 | 16.241 |
| **Benin** | 40066.34 | 28831.77 | 1.093.817 | 43013.28 | 33758.28 | 1.288.438 | 42886.21 | 33629.75 | 1.282.679 |
| **Bermuda** | 46325.68 | 35987.64 | 1.376.275 | 53295.21 | 41905.76 | 1.604.355 | 53077.07 | 42041.49 | 1.607.224 |
| **Bhutan** | 44689.2 | 34174.17 | 1.301.324 | 45675.14 | 37440 | 1.422.772 | 46332.91 | 37508.35 | 1.426.958 |
| **Bolivia (Plurinational State of)** | 46595.59 | 34970.3 | 1.335.081 | 50752.43 | 40730.04 | 1.554.107 | 51866.29 | 40593.06 | 1.548.811 |
| **Bosnia and Herzegovina** | 51630.08 | 46662.42 | 1.785.394 | 58360.25 | 46434.54 | 1.768.873 | 58870.38 | 46009.38 | 1.754.647 |
| **Botswana** | 39128.46 | 27326.72 | 1.041.038 | 46241.65 | 32530.28 | 1.247.559 | 46632.03 | 32830.2 | 1.255.154 |
| **Brazil** | 36651.43 | 24616.55 | 9.378.401 | 46108.65 | 35244.29 | 1.349.837 | 55730.74 | 43721.63 | 1.675.576 |
| **Brunei Darussalam** | 45981.57 | 33928.02 | 129.741 | 47706.15 | 39807.71 | 1.525.289 | 48397.52 | 40126.9 | 153.589 |
| **Bulgaria** | 44115.38 | 39884.8 | 1.534.919 | 50865.75 | 43525.7 | 1.659.534 | 51767.56 | 44253.74 | 1.689.317 |
| **Burkina Faso** | 39782.69 | 30385.53 | 1.156.301 | 43492.71 | 36486.32 | 1.383.987 | 43289.17 | 34158.75 | 1.296.142 |
| **Burundi** | 41734.04 | 32712.41 | 1.245.215 | 45043.17 | 35479.68 | 1.354.832 | 45441.86 | 35846 | 1.367.746 |
| **CÃ´te d'Ivoire** | 42109.53 | 30101.9 | 1.152.756 | 43498.42 | 33153.82 | 1.270.027 | 43252.65 | 32588.45 | 1.244.957 |
| **Cabo Verde** | 47458.14 | 41533.35 | 1.578.538 | 50204.57 | 41988.48 | 1.588.212 | 47730.73 | 40830.65 | 1.551.504 |
| **Cambodia** | 40137.19 | 28565.58 | 1.090.581 | 42750.79 | 33076.83 | 1.261.437 | 43092.24 | 33440.26 | 1.274.361 |
| **Cameroon** | 33283.96 | 21312.58 | 820.249 | 44757.63 | 31873.91 | 1.223.109 | 46025.02 | 32498.87 | 1.248.338 |
| **Canada** | 41767.63 | 31859.35 | 1.204.308 | 43683.48 | 34112.82 | 1.294.816 | 44160.07 | 34710.28 | 1.317.921 |
| **Central African Republic** | 39697.08 | 28748.93 | 1.091.808 | 42485.28 | 33677.68 | 1.281.679 | 42731.71 | 33149.32 | 1.262.108 |
| **Chad** | 50100.65 | 35604.57 | 1.374.911 | 47348.38 | 27733.89 | 1.070.643 | 54090.62 | 43439.59 | 1.662.672 |
| **Chile** | 43721.87 | 38701.27 | 1.484.631 | 49564.3 | 42999.87 | 1.653.061 | 50321.94 | 43266.04 | 1.663.064 |
| **China** | 23608.04 | 21872.27 | 8.408.096 | 30216.98 | 22144.82 | 8.505.873 | 32762.52 | 19990.6 | 7.691.355 |
| **Colombia** | 41794.56 | 32614.26 | 1.238.516 | 45576.7 | 35105.3 | 1.342.946 | 45501.07 | 35107.05 | 1.338.749 |
| **Comoros** | 41830.35 | 31307.73 | 118.797 | 43606.39 | 32509.11 | 1.235.738 | 44678.72 | 33276.91 | 1.267.743 |
| **Congo** | 48256.13 | 40157.79 | 1.540.157 | 48061.83 | 39895.93 | 1.530.401 | 48237.96 | 39286.45 | 1.504.252 |
| **Cook Islands** | 40485.42 | 30261.65 | 1.167.829 | 49795.26 | 36008.47 | 1.378.841 | 50172.04 | 35849.64 | 1.370.911 |
| **Costa Rica** | 51132.68 | 46196.77 | 1.780.106 | 51094.19 | 43731.9 | 1.672.641 | 52054.38 | 43938.52 | 1.678.933 |
| **Croatia** | 46835.8 | 37439.84 | 1.429.865 | 51887.36 | 41614.17 | 159.504 | 54053.23 | 43104.79 | 1.648.393 |
| **Cuba** | 31703.78 | 17450.7 | 669.327 | 37991.14 | 19420.81 | 7.450.867 | 38419.09 | 19669.44 | 7.564.893 |
| **Cyprus** | 49296.91 | 43011.55 | 1.647.442 | 51371.91 | 43661.73 | 166.797 | 51961.3 | 43740.98 | 1.671.001 |
| **Czechia** | 39809.27 | 28383.57 | 108.212 | 42717.96 | 33049.12 | 1.260.977 | 42724.25 | 33183.85 | 1.265.622 |
| **Democratic People's Republic of Korea** | 42305.72 | 36120.8 | 138.277 | 47625.08 | 41989.37 | 1.603.687 | 47799.84 | 42254.2 | 1.611.617 |
| **Democratic Republic of the Congo** | 41961.7 | 32118.17 | 1.224.406 | 44364.34 | 34924.76 | 1.325.651 | 44935.93 | 34994.15 | 1.326.577 |
| **Denmark** | 26341.6 | 12251.74 | 4.737.363 | 5.200.215 | 3.613.106 | 1.390.309 | 32981.1 | 13922.62 | 5.314.306 |
| **Djibouti** | 41346.97 | 31956.45 | 1.224.672 | 44097.83 | 33610.42 | 1.283.063 | 45170.33 | 34108.71 | 1.302.499 |
| **Dominica** | 47613.17 | 37830.09 | 1.445.416 | 53631.83 | 42790.26 | 1.645.291 | 53265.03 | 42379.11 | 1.624.352 |
| **Dominican Republic** | 46333.06 | 36746.86 | 1.401.399 | 52079.31 | 41577.62 | 1.595.566 | 52541.32 | 41761.23 | 1.600.817 |
| **Ecuador** | 47745.47 | 35366.1 | 1.358.493 | 51211.22 | 33853.81 | 1.303.349 | 52159.67 | 41032.5 | 157.388 |
| **Egypt** | 39495.14 | 29712.22 | 1.143.408 | 48763.48 | 37394.84 | 1.432.594 | 50235.85 | 38208.16 | 1.464.129 |
| **El Salvador** | 40897.61 | 30922.56 | 1.181.962 | 50059.92 | 36533.07 | 140.149 | 50064.19 | 36079.27 | 1.384.496 |
| **Equatorial Guinea** | 41247.8 | 31205.1 | 1.190.446 | 43807.84 | 30722.19 | 1.169.014 | 44253.2 | 31414.07 | 1.196.654 |
| **Eritrea** | 42133 | 32779.67 | 1.243.735 | 45282.61 | 35102.36 | 1.339.271 | 45713.99 | 35079.55 | 1.341.667 |
| **Estonia** | 47387.7 | 41846.24 | 1.605.084 | 50262.14 | 42330.96 | 1.623.538 | 51013.21 | 42870.3 | 1.641.683 |
| **Eswatini** | 39940.32 | 31478.52 | 1.207.798 | 47154.46 | 38843.91 | 1.485.038 | 47591.51 | 39172.93 | 1.499.598 |
| **Ethiopia** | 44817 | 36460.68 | 1.383.825 | 47061.44 | 37994.73 | 1.447.366 | 47173.57 | 37869.94 | 1.444.184 |
| **Fiji** | 47412.07 | 40797.2 | 155.335 | 47943.19 | 40098 | 1.524.728 | 48633.66 | 40684.68 | 1.548.054 |
| **Finland** | 31498.9 | 17331.52 | 6.663.003 | 42168.15 | 24773.72 | 9.518.016 | 42875.68 | 24955.35 | 95.552 |
| **France** | 14433.87 | 9461.45 | 3.631.933 | 42170.5 | 20095.69 | 7.749.057 | 37428.07 | 19735.21 | 7.564.824 |
| **Gabon** | 41551.66 | 31022.31 | 118.431 | 43854.96 | 31612.7 | 120.434 | 43903.11 | 31759.4 | 120.846 |
| **Gambia** | 39082.82 | 28000.52 | 1.065.844 | 42809.95 | 33650.46 | 1.283.067 | 43354.37 | 34170.04 | 1.302.441 |
| **Georgia** | 50010.82 | 44146.21 | 1.684.896 | 53254.29 | 43712.7 | 1.666.926 | 52376.4 | 43946.25 | 1.673.273 |
| **Germany** | 13309.81 | 10567.53 | 4.076.818 | 12573.78 | 11101.46 | 4.274.239 | 39229.01 | 22045.5 | 8.479.575 |
| **Ghana** | 40975.79 | 29246.36 | 1.110.892 | 43336.04 | 33654.39 | 1.279.385 | 42930.77 | 33023.16 | 1.259.562 |
| **Greece** | 18752.38 | 15388.06 | 5.919.273 | 7.101.093 | 6.053.429 | 2.343.615 | 40643.59 | 25283.08 | 9.727.372 |
| **Greenland** | 43487.81 | 28441.37 | 1.097.382 | 47009.93 | 36729.13 | 1.409.259 | 46884.25 | 37123.5 | 1.423.831 |
| **Grenada** | 45965.96 | 36432.66 | 1.385.365 | 52979.2 | 42280.05 | 1.616.793 | 53024.62 | 42229.87 | 1.619.419 |
| **Guam** | 47132.17 | 38732.88 | 1.492.643 | 47559.25 | 38830.89 | 1.485.688 | 48159.33 | 39095.18 | 1.494.971 |
| **Guatemala** | 39798.42 | 29957.13 | 1.154.148 | 49502.54 | 36384.68 | 1.394.141 | 49492.12 | 35879.9 | 1.370.381 |
| **Guinea** | 39809.48 | 28782.87 | 1.094.901 | 43224.45 | 34128.5 | 1.296.208 | 43413.29 | 34099.06 | 1.297.194 |
| **Guinea-Bissau** | 39761.92 | 28903.73 | 1.097.878 | 42910.73 | 34197.25 | 1.300.306 | 43352.35 | 34300.8 | 1.308.662 |
| **Guyana** | 47234.18 | 37788.55 | 1.444.116 | 52758.05 | 42460.91 | 1.622.764 | 52850.79 | 42526.41 | 1.625.044 |
| **Haiti** | 45083.3 | 36490.04 | 139.183 | 52055.09 | 42266.3 | 1.610.021 | 52220.29 | 42564.53 | 1.620.897 |
| **Honduras** | 39749.67 | 30240.67 | 1.165.168 | 50006.28 | 36779.88 | 1.406.711 | 49787.88 | 36438.86 | 1.397.847 |
| **Hungary** | 49107.1 | 44571.45 | 1.706.873 | 51715.18 | 44370.37 | 1.694.134 | 51828.1 | 44048.71 | 1.678.987 |
| **Iceland** | 30812.92 | 16964.36 | 6.551.619 | 37985.03 | 19292.44 | 7.406.508 | 38585.02 | 19486.5 | 7.469.951 |
| **India** | 43928.34 | 38394.72 | 1.460.109 | 46316.74 | 38194.84 | 1.456.351 | 45697.9 | 37761.57 | 144.261 |
| **Indonesia** | 49198.47 | 44109.48 | 1.681.327 | 50484.83 | 42707.07 | 1.636.274 | 50739.89 | 42985.91 | 1.647.915 |
| **Iran (Islamic Republic of)** | 47510.81 | 39930.57 | 1.529.237 | 52410.26 | 41795.7 | 1.603.936 | 54129.56 | 43267.75 | 1.660.061 |
| **Iraq** | 40400.48 | 30265.84 | 1.162.014 | 49106.98 | 37431.31 | 1.430.246 | 49658.95 | 37569.59 | 1.436.853 |
| **Ireland** | 28093.05 | 18185.79 | 7.020.821 | 36575.48 | 16745.82 | 641.757 | 37319.2 | 17060.67 | 6.539.715 |
| **Israel** | 35232.7 | 24996.94 | 9.613.586 | 47339.68 | 26807.86 | 1.028.467 | 46926.71 | 26241 | 1.007.139 |
| **Italy** | 27763.99 | 16156.14 | 6.204.132 | 38291.23 | 20116.03 | 771.787 | 44421.97 | 29967.95 | 1.149.071 |
| **Jamaica** | 47855.97 | 38008.07 | 1.455.435 | 53371.78 | 42605.61 | 16.317 | 53287.78 | 42724.14 | 1.636.244 |
| **Japan** | 44474.8 | 30720.32 | 1.178.759 | 46401.63 | 31017.43 | 1.188.977 | 47316.29 | 32910.16 | 1.261.276 |
| **Jordan** | 28747.26 | 27455.09 | 1.050.109 | 48782.57 | 36327.59 | 1.391.078 | 48890.54 | 36535.16 | 1.397.917 |
| **Kazakhstan** | 49192.64 | 42962.68 | 1.637.564 | 50434.69 | 41856.6 | 159.569 | 52208.27 | 43202.79 | 1.645.303 |
| **Kenya** | 42447.25 | 32106.12 | 1.226.759 | 46015.09 | 41147.93 | 1.574.117 | 46587.18 | 40711.86 | 1.557.701 |
| **Kiribati** | 47055.49 | 40183.36 | 153.546 | 48011.81 | 41007.85 | 1.567.383 | 47906.98 | 41328.93 | 1.578.051 |
| **Kuwait** | 31230.13 | 23936.5 | 9.151.972 | 48267.95 | 35802.62 | 1.369.628 | 49014.27 | 36942.06 | 1.417.815 |
| **Kyrgyzstan** | 49349.1 | 43228.2 | 1.649.384 | 50781.72 | 42828.97 | 1.630.865 | 52180.23 | 43952.23 | 1.671.705 |
| **Lao People's Democratic Republic** | 46851.27 | 45164.26 | 1.721.374 | 52175.6 | 50104.83 | 191.202 | 47571.2 | 40776.88 | 1.553.915 |
| **Latvia** | 44496.22 | 32665.12 | 1.247.623 | 51779.11 | 43373.2 | 1.664.917 | 51317.9 | 42917.24 | 1.648.752 |
| **Lebanon** | 39501.03 | 29454.89 | 1.131.483 | 47792.48 | 36211.55 | 138.484 | 49392.81 | 37296.23 | 1.429.998 |
| **Lesotho** | 39314.03 | 28251.2 | 1.084.525 | 46270.26 | 34217.52 | 130.711 | 46520.21 | 34356.97 | 1.309.809 |
| **Liberia** | 39644.9 | 28815.21 | 1.094.177 | 43819.23 | 35252.69 | 1.343.566 | 43742.29 | 34982.19 | 1.335.172 |
| **Libya** | 40628.61 | 29808.16 | 1.146.734 | 50263.39 | 37581.64 | 1.441.603 | 50454.28 | 38264.89 | 1.464.859 |
| **Lithuania** | 55815.53 | 44237.04 | 1.699.165 | 54308.02 | 33762.19 | 1.298.561 | 51126.92 | 43311.05 | 1.663.569 |
| **Luxembourg** | 30600.4 | 16611.04 | 6.398.976 | 38539.86 | 19420.82 | 7.452.457 | 38760.08 | 19465.64 | 7.477.246 |
| **Madagascar** | 45918.04 | 37014.42 | 1.408.298 | 45875.87 | 39584.53 | 1.513.579 | 46522.72 | 39653.37 | 1.516.833 |
| **Malawi** | 40408.86 | 31702.53 | 1.198.344 | 45332.87 | 35624.47 | 1.353.928 | 45849.84 | 35599.56 | 1.359.139 |
| **Malaysia** | 47105.21 | 38406.62 | 146.685 | 48297.63 | 40130.05 | 1.536.484 | 47430.62 | 39390.09 | 1.509.239 |
| **Maldives** | 48547.92 | 40475.18 | 1.536.663 | 46623.61 | 38560.41 | 1.472.956 | 47857.39 | 39247.86 | 1.501.186 |
| **Mali** | 39406.33 | 28422.08 | 1.085.854 | 42968.79 | 33692.23 | 1.282.307 | 42632.79 | 33651.65 | 1.277.669 |
| **Malta** | 32111.16 | 17934.76 | 6.927.642 | 38411.11 | 19690.18 | 755.633 | 38953.68 | 19764.41 | 7.598.119 |
| **Marshall Islands** | 46664.33 | 39738.21 | 15.187 | 47996.5 | 40932.61 | 1.555.797 | 48419.79 | 40883.96 | 1.551.232 |
| **Mauritania** | 40163.68 | 28739.43 | 1.091.196 | 43485.47 | 34020.53 | 1.297.834 | 43685.36 | 33742.83 | 1.287.418 |
| **Mauritius** | 46939.26 | 38908.39 | 1.485.156 | 48788.41 | 40113.7 | 1.532.427 | 47951.4 | 39300.38 | 1.505.471 |
| **Mexico** | 39417.2 | 28839.09 | 1.106.941 | 53103.87 | 41866.14 | 16.067 | 52731.36 | 41186.02 | 1.579.765 |
| **Micronesia (Federated States of)** | 47837.53 | 40947.1 | 156.454 | 48497.47 | 41291.57 | 1.569.761 | 48593.93 | 41188.68 | 1.570.844 |
| **Monaco** | 29985.71 | 16032.93 | 6.166.196 | 38139.05 | 18636.75 | 7.167.346 | 38417.82 | 18626.25 | 7.137.436 |
| **Mongolia** | 48806.14 | 42161.44 | 1.603.303 | 48838.06 | 40841.15 | 1.558.997 | 51343.18 | 42794.36 | 1.635.589 |
| **Montenegro** | 49270.98 | 42783.1 | 1.644.945 | 51469.74 | 44101.88 | 1.691.856 | 51517.01 | 44406.83 | 1.707.115 |
| **Morocco** | 39779.77 | 30089.75 | 1.153.232 | 49099.21 | 37810.39 | 1.448.937 | 50200.12 | 38625.65 | 1.477.783 |
| **Mozambique** | 41320.64 | 32753.7 | 124.288 | 45229.86 | 35296.28 | 1.343.723 | 45101.73 | 35118.05 | 133.972 |
| **Myanmar** | 34413.94 | 29237.26 | 1.114.449 | 24921.85 | 23133.73 | 8.741.243 | 45550.75 | 31344.9 | 1.187.274 |
| **Namibia** | 35687.38 | 23323.09 | 8.905.418 | 46056.66 | 32498.9 | 124.308 | 46188.39 | 32765.02 | 1.252.572 |
| **Nauru** | 45810.26 | 38361.41 | 1.471.058 | 47214.75 | 40403.66 | 15.486 | 48002.08 | 39996.03 | 1.530.213 |
| **Nepal** | 45621.64 | 36445.41 | 1.390.247 | 51768.53 | 41177.36 | 1.572.903 | 51821.39 | 43192.52 | 1.659.381 |
| **Netherlands** | 35809.12 | 22395.91 | 8.659.084 | 37008.63 | 19870.77 | 7.630.352 | 43594.29 | 21027.71 | 8.077.036 |
| **New Zealand** | 26271.62 | 14341.17 | 5.490.451 | 10725.35 | 9.175.353 | 354.235 | 11602.72 | 9.968.406 | 3.812.617 |
| **Nicaragua** | 40528.23 | 30778.43 | 1.171.671 | 49813.3 | 37024.07 | 141.791 | 50386.45 | 36819.48 | 1.410.654 |
| **Niger** | 39974.62 | 31191.33 | 1.190.336 | 44761.56 | 40482.21 | 1.541.312 | 44687.11 | 40376.83 | 1.537.476 |
| **Nigeria** | 31332.58 | 22889.25 | 8.679.911 | 41185.49 | 25615.85 | 9.725.291 | 42805.15 | 28304.7 | 1.075.756 |
| **Niue** | 48388.44 | 40651.72 | 1.552.979 | 48963.07 | 40543.59 | 1.553.598 | 48297.93 | 40464.21 | 1.550.752 |
| **North Macedonia** | 49187.71 | 42683.84 | 1.639.024 | 51075.59 | 43597.73 | 1.669.849 | 51512.63 | 44075.55 | 169.079 |
| **Northern Mariana Islands** | 40393.16 | 32165.38 | 122.876 | 48337.26 | 39640.29 | 1.521.047 | 47753.01 | 39136.96 | 1.500.067 |
| **Norway** | 23641.51 | 11494.38 | 4.417.411 | 43963.7 | 28382.72 | 1.087.258 | 45264.88 | 29801 | 1.141.729 |
| **Oman** | 42939.54 | 32311.43 | 1.237.337 | 48958.04 | 38053.37 | 1.456.274 | 50369.09 | 39262.4 | 150.454 |
| **Pakistan** | 43379.61 | 41845.78 | 1.590.186 | 41445.33 | 37402.28 | 1.428.472 | 51315.93 | 42200.66 | 1.612.686 |
| **Palau** | 47530.96 | 39759.71 | 1.532.571 | 48011.72 | 40379.52 | 154.732 | 48691.51 | 40919.36 | 1.568.878 |
| **Palestine** | 38672.59 | 27089.94 | 1.038.001 | 46720.97 | 33076.83 | 1.262.542 | 51060.49 | 34627.38 | 1.327.208 |
| **Panama** | 40172.05 | 29961.35 | 1.151.161 | 49785.59 | 35961.18 | 1.375.983 | 49875.79 | 35653.52 | 1.367.787 |
| **Papua New Guinea** | 46091.2 | 39314.43 | 1.498.277 | 46887.71 | 39675.33 | 1.507.505 | 47200.05 | 40269.07 | 1.530.954 |
| **Paraguay** | 43559.67 | 33290.49 | 1.273.447 | 52837.66 | 39465.52 | 1.510.469 | 52993.64 | 39470.11 | 1.509.461 |
| **Peru** | 46508.64 | 34453.35 | 131.558 | 51291.22 | 40695.44 | 1.559.454 | 51916.8 | 40700.46 | 1.556.706 |
| **Philippines** | 49607.17 | 45327.8 | 1.728.118 | 49117.05 | 41278 | 1.581.001 | 49532.15 | 41517.72 | 1.589.827 |
| **Poland** | 51724.6 | 47867.93 | 1.837.336 | 52922.97 | 43171.79 | 1.657.637 | 53387.7 | 43642.59 | 1.673.979 |
| **Portugal** | 29373.47 | 17101.46 | 6.615.408 | 37640.2 | 19664.63 | 7.521.573 | 39148.61 | 20010.01 | 7.676.223 |
| **Puerto Rico** | 47192.2 | 36796.29 | 1.414.773 | 53724.46 | 42185.44 | 1.613.839 | 53444.2 | 42192.92 | 1.614.586 |
| **Qatar** | 40122.08 | 31124.77 | 1.193.832 | 53098.61 | 46424.36 | 178.417 | 51305.59 | 40792.43 | 1.563.515 |
| **Republic of Korea** | 49501.25 | 43331.63 | 1.657.309 | 51536.38 | 45456.28 | 1.746.948 | 49510.09 | 42569.26 | 162.973 |
| **Republic of Moldova** | 48649.52 | 44249.45 | 1.690.869 | 50832.49 | 43605.3 | 1.676.307 | 51479.06 | 44144.04 | 1.691.375 |
| **Romania** | 52024.66 | 54232.12 | 2.084.441 | 51708.57 | 44128.18 | 1.691.607 | 52141.59 | 44499.88 | 1.706.767 |
| **Russian Federation** | 47177.16 | 41956.9 | 1.608.855 | 52428.83 | 43679.37 | 1.675.408 | 54268.96 | 45181 | 1.733.197 |
| **Rwanda** | 41956.3 | 29079.41 | 1.110.319 | 45378.95 | 35394.57 | 134.732 | 45463.5 | 34703.53 | 1.327.532 |
| **Saint Kitts and Nevis** | 46971.41 | 36878.98 | 1.406.469 | 53315.32 | 42109.41 | 1.617.225 | 53134.79 | 42020.7 | 1.607.521 |
| **Saint Lucia** | 47607.31 | 38091.14 | 1.460.013 | 53324.64 | 42590.3 | 1.629.311 | 53302.52 | 42534.41 | 1.629.194 |
| **Saint Vincent and the Grenadines** | 47172.24 | 40925.99 | 1.568.816 | 53726.67 | 44767.86 | 1.716.855 | 53578.34 | 44894.51 | 1.715.734 |
| **Samoa** | 46586.3 | 39787.85 | 1.517.396 | 50040.42 | 42087.98 | 1.603.466 | 49597.29 | 41998.48 | 1.597.022 |
| **San Marino** | 30157.94 | 16341.14 | 6.304.554 | 38864.15 | 19450.17 | 7.469.471 | 39136.08 | 19526.59 | 7.470.763 |
| **Sao Tome and Principe** | 40159.99 | 28806.67 | 1.107.402 | 44125.56 | 34273.9 | 1.309.835 | 43760.76 | 33764.47 | 1.292.379 |
| **Saudi Arabia** | 21997.31 | 19550.91 | 7.460.145 | 40636.46 | 27891.26 | 1.073.939 | 44263.47 | 33448.04 | 1.285.336 |
| **Senegal** | 40160.26 | 28624.42 | 1.087.989 | 47110.46 | 36420.72 | 1.385.962 | 40667.5 | 34006.31 | 1.298.817 |
| **Serbia** | 46756.37 | 45449.49 | 1.732.998 | 54824.94 | 51295.07 | 1.964.713 | 52294.13 | 45307.01 | 1.732.636 |
| **Seychelles** | 47037.09 | 38766.4 | 1.477.621 | 47482.44 | 38762.76 | 1.486.853 | 47940.93 | 39293.67 | 1.509.873 |
| **Sierra Leone** | 41956.63 | 30570.69 | 1.161.237 | 41766.84 | 30914.93 | 1.173.994 | 41920.45 | 31151.76 | 1.183.858 |
| **Singapore** | 22332.82 | 17257.72 | 6.611.304 | 36885.46 | 19834.79 | 763.321 | 48026.43 | 37808.71 | 1.451.201 |
| **Slovakia** | 49957.72 | 44125.4 | 1.696.977 | 50469.38 | 42895.82 | 1.640.141 | 51318.54 | 43736.52 | 1.673.773 |
| **Slovenia** | 49844.19 | 42751.86 | 1.639.227 | 50195.88 | 42464.6 | 1.620.869 | 51985.96 | 43788.95 | 1.672.719 |
| **Solomon Islands** | 45791.63 | 38842.3 | 1.484.289 | 47083.76 | 40672.54 | 1.552.437 | 47470.07 | 40849.78 | 1.562.211 |
| **Somalia** | 40894.02 | 32994.62 | 1.260.736 | 45177.48 | 36772.49 | 1.401.064 | 45194.02 | 36865.82 | 1.406.247 |
| **South Africa** | 35297.42 | 22370.23 | 8.561.125 | 48555.09 | 37178.52 | 1.425.388 | 49308.05 | 37555.66 | 1.439.542 |
| **South Sudan** | 41024.18 | 31308.01 | 1.180.022 | 44078.21 | 32458.74 | 1.235.125 | 43740.9 | 32275.94 | 1.227.754 |
| **Spain** | 14763.06 | 11037.41 | 4.237.173 | 40029.99 | 23999.88 | 9.198.888 | 40427.38 | 24055.37 | 9.233.586 |
| **Sri Lanka** | 46681.47 | 28064.59 | 1.071.754 | 51483.43 | 33178.75 | 1.278.444 | 48436.2 | 39441.47 | 1.515.577 |
| **Sudan** | 19731.83 | 15955.31 | 6.115.788 | 30977.51 | 25918.22 | 9.943.043 | 48713.99 | 35390.07 | 1.350.497 |
| **Suriname** | 47374.13 | 40466.29 | 1.552.626 | 52382.34 | 41763.83 | 1.600.788 | 53400.83 | 42427.5 | 162.354 |
| **Sweden** | 20840.78 | 13889.64 | 5.329.627 | 32735.15 | 27587.94 | 1.063.333 | 44749.43 | 32647.16 | 1.253.452 |
| **Switzerland** | 10204.68 | 7525.33 | 2.892.674 | 16911.59 | 9.009.468 | 3.456.817 | 37796.55 | 17675.17 | 6.775.676 |
| **Syrian Arab Republic** | 40695.34 | 28595.33 | 1.097.419 | 53956.75 | 32213.47 | 123.719 | 48997.71 | 32911.6 | 1.257.271 |
| **Taiwan (Province of China)** | 45519.26 | 35128.07 | 1.342.762 | 52470.44 | 46473.86 | 1.777.885 | 48986.96 | 42228.18 | 1.616.141 |
| **Tajikistan** | 48589.95 | 42473.51 | 1.623.707 | 50484.33 | 42549.73 | 1.618.792 | 51295.68 | 43551.07 | 1.658.867 |
| **Thailand** | 51479.39 | 41156.63 | 1.566.851 | 57768.84 | 42308.19 | 1.620.816 | 48798.68 | 41288.34 | 1.574.916 |
| **Timor-Leste** | 46375.38 | 37836.04 | 1.438.953 | 47443.07 | 39603.44 | 1.500.194 | 47407.56 | 39787.88 | 150.833 |
| **Togo** | 40227.59 | 29093.3 | 1.111.308 | 43402.14 | 34333.18 | 1.310.295 | 43791.01 | 34625.27 | 1.319.714 |
| **Tokelau** | 46890.97 | 40139.29 | 1.543.092 | 47297.7 | 39722.07 | 1.522.163 | 47919.7 | 40133.79 | 1.537.879 |
| **Tonga** | 46538.2 | 39280.06 | 1.501.635 | 48726.1 | 41128.61 | 1.579.309 | 48946.96 | 41381.88 | 158.426 |
| **Trinidad and Tobago** | 46417.93 | 32217.18 | 1.233.927 | 40817.69 | 27637.08 | 1.064.619 | 41265.53 | 27881.74 | 1.073.088 |
| **Tunisia** | 41249.39 | 30957.57 | 1.184.536 | 49184.53 | 37568.42 | 1.443.064 | 50127.91 | 38146.45 | 1.464.707 |
| **Turkey** | 43319.51 | 30784.23 | 1.172.518 | 54017.82 | 38078.66 | 1.465.967 | 49533.93 | 37572.91 | 1.441.568 |
| **Turkmenistan** | 48834.37 | 42171.96 | 1.607.375 | 50505.95 | 42087.22 | 161.263 | 51636.28 | 42864.1 | 1.641.395 |
| **Tuvalu** | 46914.58 | 40234.65 | 153.482 | 48691.57 | 41560.63 | 1.589.287 | 48435.96 | 41163.73 | 157.545 |
| **Uganda** | 31020.21 | 28713.92 | 1.088.099 | 49589.73 | 28971.35 | 1.106.424 | 45298.98 | 31927.17 | 1.217.931 |
| **Ukraine** | 50057.58 | 45999.91 | 1.765.819 | 53065.06 | 43859.4 | 1.687.967 | 54510.38 | 45333.78 | 1.737.214 |
| **United Arab Emirates** | 43194.99 | 30854.76 | 1.187.469 | 52678.91 | 38873.14 | 1.492.732 | 58644.24 | 42481.49 | 1.632.351 |
| **United Kingdom** | 22223.82 | 13027.9 | 5.006.779 | 13882.46 | 12243.65 | 4.708.387 | 15766.83 | 12227.71 | 4.686.853 |
| **United Republic of Tanzania** | 29463.48 | 13711.74 | 5.226.015 | 20249.88 | 13753.08 | 527.034 | 17107.87 | 12869.82 | 4.947.282 |
| **United States of America** | 46104.26 | 35847.96 | 1.368.358 | 53096.24 | 41570.22 | 1.591.702 | 53176.37 | 41787.29 | 1.601.164 |
| **United States Virgin Islands** | 23685.9 | 13249.85 | 5.099.394 | 42990.07 | 27582.92 | 1.058.034 | 49103.54 | 38848.08 | 1.489.595 |
| **Uruguay** | 46234.84 | 36274.52 | 1.389.754 | 44870.38 | 32741.87 | 1.255.365 | 49738.81 | 37111.81 | 1.424.938 |
| **Uzbekistan** | 49222.41 | 43141.04 | 1.644.415 | 51029.68 | 42690.57 | 1.619.443 | 51927.04 | 43534.21 | 1.653.993 |
| **Vanuatu** | 46042.48 | 39153.8 | 1.498.265 | 48195.36 | 40964.6 | 1.560.069 | 48544.68 | 41027.68 | 1.563.509 |
| **Venezuela (Bolivarian Republic of)** | 40135.35 | 29694.95 | 1.141.573 | 49492.27 | 35570.4 | 1.360.394 | 49396.91 | 35751 | 1.369.021 |
| **Viet Nam** | 47656.21 | 40695.16 | 155.809 | 47833.04 | 40335.31 | 1.538.908 | 53624.82 | 43142.27 | 1.648.584 |
| **Yemen** | 39328.01 | 29957.48 | 1.140.195 | 48886.24 | 37888.16 | 1.434.943 | 49600.83 | 38702.91 | 1.466.663 |
| **Zambia** | 41031.11 | 32187.47 | 1.218.389 | 44824.36 | 34117.58 | 1.300.451 | 44883.18 | 33919.53 | 1.291.078 |
| **Zimbabwe** | 27252.42 | 20827.61 | 7.991.468 | 43464.93 | 27687.33 | 1.059.717 | 44725.42 | 28117.5 | 1.076.445 |

Rate per 100.00 thousand / Source: Global Burden of Disease (GBD)
