# Supplementary material for: Structural determinants of inequalities in untreated dental caries in the Global Burden of Disease Study
Source: PLoS One. 2025 Jun 3;20(6):e0325138. doi: 10.1371/journal.pone.0325138 (PMC12132969; doi:10.1371/journal.pone.0325138)
Supplement: S3 Appendix C — (DOCX) [file pone.0325138.s003.docx]

Appendix C – Prevalence, incidence and YLDs of caries in permanent dentition in 2000, 2010 and 2019 by country.

|  | Caries in permanent teeth 15 – 49 years / 2000 | | | Caries in permanent teeth 15 – 49 years / 2010 | | | Caries in permanent teeth 15 – 49 years / 2019 | | |
| --- | --- | --- | --- | --- | --- | --- | --- | --- | --- |
| **Countries** | Incidence | Prevalence | YLDs | Incidence | Prevalence | YLDs | Incidence | Prevalence | YLDs |
| **Afghanistan** | 48087.18 | 42321.44 | 415.614 | 48766.95 | 41507.03 | 4.095.692 | 48766.95 | 40806.21 | 4.031.096 |
| **Albania** | 41583.02 | 47898.73 | 4.765.534 | 43903.54 | 46572.66 | 4.639.818 | 43903.54 | 45889.69 | 4.565.074 |
| **Algeria** | 48299.9 | 38710.26 | 3.860.354 | 49413.04 | 38693.51 | 385.477 | 49413.04 | 38285.2 | 380.638 |
| **American Samoa** | 50069.45 | 36187.24 | 3.597.788 | 50172.28 | 36540.4 | 363.329 | 50172.28 | 36517.55 | 3.634.869 |
| **Andorra** | 51074.41 | 34835.76 | 3.457.085 | 50123.3 | 34102.09 | 3.383.871 | 50123.3 | 34277.18 | 3.395.477 |
| **Angola** | 51701.63 | 35573.75 | 3.514.842 | 52030.59 | 34519.4 | 3.420.485 | 52030.59 | 34124.48 | 3.382.854 |
| **Antigua and Barbuda** | 49329.24 | 33269.45 | 3.305.626 | 49345.41 | 32656.18 | 324.414 | 49345.41 | 32507.42 | 3.228.724 |
| **Argentina** | 47922.28 | 39380.7 | 3.912.728 | 47773.48 | 39578.12 | 3.931.945 | 47773.48 | 39187.28 | 3.889.153 |
| **Armenia** | 48128.82 | 39702.62 | 3.954.497 | 49142.43 | 38635.18 | 3.851.322 | 49142.43 | 37735.55 | 3.759.339 |
| **Australia** | 52080.18 | 29814.7 | 2.953.001 | 51705.68 | 30745.97 | 3.038.633 | 51705.68 | 34004.86 | 3.359.934 |
| **Austria** | 50156.91 | 32539.25 | 3.225.985 | 51088.74 | 32190.46 | 3.193.108 | 51088.74 | 32051.11 | 317.919 |
| **Azerbaijan** | 48939.31 | 39796.7 | 3.966.352 | 49862.05 | 38444.56 | 3.834.311 | 49862.05 | 37413.79 | 3.726.968 |
| **Bahamas** | 49502.98 | 32587.61 | 3.244.245 | 49652.78 | 32470.97 | 3.225.055 | 49652.78 | 32583.44 | 3.236.627 |
| **Bahrain** | 49059.72 | 36947.63 | 3.673.092 | 47825.22 | 36618.73 | 3.642.398 | 47825.22 | 36424.32 | 3.616.992 |
| **Bangladesh** | 53405.9 | 33186.4 | 3.302.335 | 53879.58 | 32526.52 | 323.746 | 53879.58 | 31813.5 | 3.165.637 |
| **Barbados** | 48857.9 | 33391.49 | 3.320.426 | 48989.13 | 33095.45 | 3.290.833 | 48989.13 | 33092.24 | 3.291.362 |
| **Belarus** | 44905.05 | 43472.99 | 4.316.482 | 46480.07 | 42424.7 | 4.214.272 | 46480.07 | 41732.82 | 4.143.792 |
| **Belgium** | 50541.88 | 30287.28 | 3.002.273 | 50864.4 | 30239.43 | 299.132 | 50864.4 | 30109.55 | 2.982.967 |
| **Belize** | 49369.52 | 34578.3 | 3.442.592 | 49139.61 | 34447.05 | 3.425.067 | 49139.61 | 33947.26 | 3.377.231 |
| **Benin** | 49597.38 | 38047.79 | 3.777.185 | 49423.88 | 37649.02 | 3.741.838 | 49423.88 | 36740.12 | 3.655.942 |
| **Bermuda** | 48886.49 | 32282.62 | 3.210.581 | 48908.08 | 31719.51 | 3.151.305 | 48908.08 | 31361.41 | 3.116.782 |
| **Bhutan** | 53003.09 | 32244.74 | 3.213.784 | 54419.77 | 31706.95 | 3.162.051 | 54419.77 | 31181.48 | 3.104.634 |
| **Bolivia (Plurinational State of)** | 46487.51 | 41223.93 | 4.101.087 | 47063.52 | 40696.25 | 4.052.471 | 47063.52 | 40122.3 | 3.993.526 |
| **Bosnia and Herzegovina** | 42842.23 | 44350.47 | 4.407.272 | 44376.35 | 43355.01 | 4.309.285 | 44376.35 | 42951.2 | 4.261.506 |
| **Botswana** | 49242.88 | 37556.34 | 3.706.557 | 49211.85 | 37490.92 | 3.701.221 | 49211.85 | 37831.96 | 3.728.658 |
| **Brazil** | 51510.13 | 29931.44 | 2.969.895 | 51831.94 | 28063.57 | 2.786.304 | 51831.94 | 29417.4 | 2.918.633 |
| **Brunei Darussalam** | 51244.28 | 32024.18 | 3.194.223 | 51539.78 | 31953.13 | 3.189.367 | 51539.78 | 32110.28 | 3.198.364 |
| **Bulgaria** | 42443.02 | 46282.5 | 460.078 | 43867.04 | 45330.22 | 4.506.767 | 43867.04 | 44616.72 | 4.431.624 |
| **Burkina Faso** | 48158.68 | 38853.15 | 3.852.057 | 50625.26 | 35110.42 | 349.022 | 50625.26 | 34668.52 | 3.453.897 |
| **Burundi** | 49027.58 | 38493.1 | 3.792.875 | 50004.18 | 38340.3 | 3.784.764 | 50004.18 | 38067.7 | 3.763.816 |
| **CÃ´te d'Ivoire** | 49352.96 | 36641.82 | 3.650.519 | 50273 | 36046.85 | 3.589.375 | 50.273 | 35246.07 | 3.511.928 |
| **Cabo Verde** | 51398.23 | 35456.04 | 352.117 | 52672.17 | 34644.23 | 3.452.848 | 52672.17 | 33413.22 | 3.331.775 |
| **Cambodia** | 49636.84 | 37255.48 | 3.692.312 | 50120.32 | 36936.8 | 3.670.415 | 50120.32 | 36551.11 | 3.634.932 |
| **Cameroon** | 51240.97 | 29306.83 | 2.914.717 | 52163.54 | 29199.51 | 2.904.922 | 52163.54 | 29227.26 | 2.900.363 |
| **Canada** | 51206.45 | 36747.18 | 3.622.723 | 50484.74 | 36772.3 | 3.634.959 | 50484.74 | 36831.1 | 3.642.176 |
| **Central African Republic** | 48965.31 | 38475.27 | 3.819.443 | 50351.51 | 37651.52 | 3.740.897 | 50351.51 | 37302.09 | 3.708.701 |
| **Chad** | 35687.39 | 57377.14 | 5.688.059 | 40208.53 | 42584.59 | 4.231.436 | 40208.53 | 52791.19 | 5.240.506 |
| **Chile** | 52924.8 | 29442.2 | 2.941.934 | 52367.55 | 28303.59 | 2.830.033 | 52367.55 | 28365.62 | 2.833.221 |
| **China** | 47313.16 | 37312.91 | 3.711.987 | 40280.9 | 48888.93 | 4.863.408 | 40280.9 | 48814.22 | 4.851.517 |
| **Colombia** | 50127.95 | 37552.68 | 373.317 | 49991.21 | 36953.4 | 3.673.726 | 49991.21 | 37245.34 | 3.703.585 |
| **Comoros** | 51651.04 | 34712.85 | 3.429.052 | 51035.07 | 34982.7 | 3.460.012 | 51035.07 | 34533.53 | 3.417.983 |
| **Congo** | 50315.82 | 36259.06 | 3.613.896 | 50046.53 | 35646.18 | 3.553.722 | 50046.53 | 35510.26 | 3.530.965 |
| **Cook Islands** | 47347.89 | 34157.51 | 3.401.503 | 48255.31 | 33631.3 | 3.346.134 | 48255.31 | 32951.63 | 3.277.295 |
| **Costa Rica** | 42090.71 | 47647.39 | 4.734.889 | 42366.94 | 47513.66 | 4.721.508 | 42366.94 | 47074.9 | 4.675.193 |
| **Croatia** | 47443.23 | 38453.8 | 3.816.676 | 47683.43 | 42635 | 4.235.258 | 47683.43 | 36841.39 | 3.658.958 |
| **Cuba** | 51382.42 | 35827.81 | 3.559.641 | 51010.73 | 35126.86 | 3.491.711 | 51010.73 | 35319.11 | 3.503.036 |
| **Cyprus** | 43914.94 | 45271.46 | 4.502.068 | 44063.04 | 44365.5 | 440.635 | 44063.04 | 43808.06 | 4.346.886 |
| **Czechia** | 49844.02 | 37084.81 | 3.672.285 | 50391.18 | 36795.81 | 3.647.395 | 50391.18 | 36131.2 | 3.586.731 |
| **Democratic People's Republic of Korea** | 52500.77 | 29582.91 | 2.954.733 | 52754.37 | 29085.49 | 2.906.433 | 52754.37 | 29725.3 | 2.971.499 |
| **Democratic Republic of the Congo** | 50482.59 | 37113.33 | 3.651.868 | 51406.27 | 37256.72 | 367.885 | 51406.27 | 36777.19 | 3.637.001 |
| **Denmark** | 50362.87 | 28308.38 | 280.168 | 50487.75 | 26864.04 | 2.660.473 | 50487.75 | 27800.72 | 2.758.004 |
| **Djibouti** | 50551.29 | 36957.1 | 3.671.709 | 49910.71 | 36674.97 | 364.362 | 49910.71 | 35914.02 | 3.565.042 |
| **Dominica** | 48946.87 | 34260.83 | 3.408.398 | 49565.91 | 33825.67 | 3.361.502 | 49565.91 | 33795.76 | 3.354.578 |
| **Dominican Republic** | 49440.15 | 34557.46 | 3.438.567 | 50103.89 | 34231.91 | 3.404.708 | 50103.89 | 33616.54 | 3.344.913 |
| **Ecuador** | 45656.87 | 42935.58 | 4.279.204 | 47515.36 | 39649.68 | 3.951.525 | 47515.36 | 39401.98 | 3.924.604 |
| **Egypt** | 49663.55 | 34824.45 | 3.467.402 | 50101.2 | 34464.03 | 3.432.047 | 50101.2 | 33717.09 | 3.358.644 |
| **El Salvador** | 47671.72 | 35051.8 | 3.481.015 | 48229.93 | 34370.88 | 3.416.539 | 48229.93 | 33928.91 | 3.376.591 |
| **Equatorial Guinea** | 52115.65 | 34379.91 | 3.396.211 | 53658.74 | 32054.72 | 3.177.529 | 53658.74 | 32290.78 | 3.206.378 |
| **Eritrea** | 48835.64 | 40586.88 | 400.321 | 48312.72 | 40326.83 | 3.992.167 | 48312.72 | 40408.43 | 4.009.911 |
| **Estonia** | 47319.15 | 38232.92 | 3.799.607 | 48252.57 | 37316.74 | 3.714.449 | 48252.57 | 36485.37 | 3.625.826 |
| **Eswatini** | 45942.11 | 45726.78 | 4.518.818 | 45825.64 | 45431.55 | 4.479.111 | 45825.64 | 45314.54 | 446.677 |
| **Ethiopia** | 52554.48 | 34018.87 | 33.695 | 53571.79 | 33610.2 | 3.336.202 | 53571.79 | 33078.87 | 3.286.553 |
| **Fiji** | 49731.37 | 38146.33 | 379.216 | 49818.46 | 37570.77 | 3.736.135 | 49818.46 | 37517.96 | 3.726.065 |
| **Finland** | 51064.64 | 30553.24 | 3.025.551 | 51253.68 | 32653.28 | 323.201 | 51253.68 | 32031.6 | 317.596 |
| **France** | 49053.4 | 38899.25 | 3.856.183 | 49119.55 | 38678.55 | 3.836.267 | 49119.55 | 38138.11 | 3.785.921 |
| **Gabon** | 52223.31 | 33316.8 | 3.294.376 | 52081.23 | 33340.79 | 3.298.357 | 52081.23 | 33234.52 | 3.288.082 |
| **Gambia** | 49889.52 | 38051.01 | 3.775.713 | 49750.49 | 37746.48 | 3.743.153 | 49750.49 | 37259.7 | 3.700.808 |
| **Georgia** | 47112.61 | 41504.63 | 4.136.946 | 48005.44 | 41927.14 | 417.338 | 48005.44 | 39660.56 | 3.944.704 |
| **Germany** | 50650.85 | 35072.52 | 3.477.787 | 51638.94 | 33675.63 | 3.335.941 | 51638.94 | 33519.08 | 3.324.819 |
| **Ghana** | 49823.12 | 31656.01 | 3.141.932 | 50569.38 | 31527.1 | 3.134.385 | 50569.38 | 30789.41 | 3.064.062 |
| **Greece** | 49781.3 | 38861.42 | 3.855.605 | 49604.82 | 38010.08 | 3.769.999 | 49604.82 | 38580.21 | 3.821.454 |
| **Greenland** | 51518.17 | 29112.13 | 2.871.847 | 53299.88 | 28614.16 | 2.829.424 | 53299.88 | 29108.79 | 2.882.639 |
| **Grenada** | 50014.38 | 34409.77 | 3.420.909 | 48845.84 | 33625.39 | 3.343.974 | 48845.84 | 33234.71 | 3.303.714 |
| **Guam** | 50169.02 | 34936.13 | 3.484.105 | 51061.61 | 35224.36 | 3.509.217 | 51061.61 | 34816.07 | 3.465.735 |
| **Guatemala** | 47925.15 | 35200.23 | 349.753 | 49105.44 | 34582.55 | 3.440.059 | 49105.44 | 34296.12 | 3.410.719 |
| **Guinea** | 49220.46 | 38231.17 | 3.798.035 | 49947.84 | 37858.59 | 3.761.002 | 49947.84 | 37352.87 | 3.712.265 |
| **Guinea-Bissau** | 47469.07 | 42332.73 | 4.201.578 | 47822.19 | 41965.64 | 4.169.281 | 47822.19 | 41693.77 | 4.143.807 |
| **Guyana** | 48847.05 | 35273.32 | 3.488.602 | 49509.07 | 34640.13 | 3.433.651 | 49509.07 | 34305.27 | 3.399.746 |
| **Haiti** | 48708.73 | 36758.27 | 3.637.983 | 48790.05 | 36437.77 | 3.613.728 | 48790.05 | 36373.13 | 3.598.663 |
| **Honduras** | 47195.04 | 35591.72 | 3.544.614 | 47958.29 | 35368.71 | 3.526.978 | 47958.29 | 34555.89 | 3.444.163 |
| **Hungary** | 42447.24 | 45795.12 | 455.023 | 43888.83 | 44559.09 | 4.433.313 | 43888.83 | 44114.94 | 438.301 |
| **Iceland** | 45211.49 | 46995.9 | 4.666.652 | 47976.39 | 43584.9 | 4.327.532 | 47976.39 | 41647.79 | 4.136.519 |
| **India** | 54230.59 | 31421.37 | 3.110.654 | 54115.49 | 31053.45 | 308.133 | 54115.49 | 30812.34 | 305.616 |
| **Indonesia** | 53617.35 | 32107.78 | 3.197.173 | 53099.57 | 31751.41 | 3.165.017 | 53099.57 | 31166.28 | 3.105.095 |
| **Iran (Islamic Republic of)** | 50954.55 | 36353.01 | 3.616.549 | 50407.48 | 37344.27 | 3.712.477 | 50407.48 | 36028.86 | 3.572.725 |
| **Iraq** | 48838.06 | 39471.3 | 3.914.986 | 49982.93 | 38814.84 | 3.853.678 | 49982.93 | 38161.73 | 3.785.781 |
| **Ireland** | 52042.95 | 34854.01 | 3.457.241 | 51278.86 | 33756.24 | 3.346.706 | 51278.86 | 33921.93 | 3.360.044 |
| **Israel** | 50835.24 | 38402.59 | 3.816.444 | 50922.03 | 37891.16 | 3.767.094 | 50922.03 | 37803.5 | 3.754.758 |
| **Italy** | 51969.64 | 32193.53 | 3.190.856 | 51391.49 | 31601.1 | 3.134.738 | 51391.49 | 31491.94 | 3.121.682 |
| **Jamaica** | 49367.41 | 34640.28 | 344.439 | 49429.09 | 34134.81 | 3.396.424 | 49429.09 | 34119.4 | 3.393.966 |
| **Japan** | 52369.54 | 30305.29 | 3.018.815 | 51320.14 | 30184.33 | 3.005.341 | 51320.14 | 29777.35 | 2.963.971 |
| **Jordan** | 48253.31 | 40425.85 | 4.030.281 | 47906.65 | 41308.69 | 4.117.794 | 47906.65 | 41129.31 | 4.096.056 |
| **Kazakhstan** | 48743.61 | 35657.34 | 3.546.517 | 49202.1 | 36949.62 | 3.678.077 | 49202.1 | 35706.34 | 3.555.558 |
| **Kenya** | 53796.9 | 29497.83 | 2.924.745 | 53292.07 | 29636.25 | 2.945.505 | 53292.07 | 29155.24 | 2.898.555 |
| **Kiribati** | 48496.57 | 39337.68 | 3.905.795 | 49488.88 | 39678.62 | 3.942.709 | 49488.88 | 39551.42 | 3.927.733 |
| **Kuwait** | 50476.55 | 36590.64 | 3.636.113 | 49151.38 | 36004.72 | 3.583.407 | 49151.38 | 35719.66 | 3.542.988 |
| **Kyrgyzstan** | 48243.01 | 40550.2 | 4.042.072 | 48750.1 | 40366.89 | 4.027.516 | 48750.1 | 40149.01 | 4.004.808 |
| **Lao People's Democratic Republic** | 52711.65 | 32270.58 | 3.216.539 | 52938.66 | 32278.34 | 322.295 | 52938.66 | 30873.79 | 3.081.297 |
| **Latvia** | 45891.16 | 42633.84 | 4.233.224 | 46838.78 | 41496.62 | 4.126.304 | 46838.78 | 40360.98 | 4.005.758 |
| **Lebanon** | 48459 | 38802.86 | 3.850.267 | 49559.21 | 38002.95 | 3.773.216 | 49559.21 | 37887.41 | 3.761.625 |
| **Lesotho** | 48455.54 | 39.989 | 3.954.351 | 48649.9 | 39673.13 | 3.916.996 | 48649.9 | 39383.85 | 3.881.735 |
| **Liberia** | 48676.67 | 39770.49 | 3.912.978 | 49720.77 | 39074.84 | 3.856.966 | 49720.77 | 38440.3 | 3.799.379 |
| **Libya** | 49226.05 | 37549.46 | 3.740.089 | 48459.91 | 37409.75 | 371.737 | 48459.91 | 38327.54 | 3.800.914 |
| **Lithuania** | 42919 | 47194.06 | 46.883 | 46692.38 | 44399.42 | 441.096 | 46692.38 | 41736.65 | 4.145.518 |
| **Luxembourg** | 51223.45 | 34943.49 | 3.459.519 | 51650.98 | 34338.89 | 3.404.454 | 51650.98 | 34187.79 | 3.388.689 |
| **Madagascar** | 47051.33 | 43931.8 | 4.353.661 | 47376.37 | 43557.69 | 4.326.923 | 47376.37 | 43216.14 | 4.294.078 |
| **Malawi** | 49861.2 | 38372.8 | 3.794.855 | 50375.55 | 37746.66 | 374.347 | 50375.55 | 37470.11 | 3.721.425 |
| **Malaysia** | 50890 | 26556.85 | 2.645.865 | 51531.62 | 26865.27 | 2.680.219 | 51531.62 | 26120.23 | 2.603.562 |
| **Maldives** | 53545.39 | 32358.22 | 3.226.571 | 52975.15 | 31387.08 | 3.138.497 | 52975.15 | 30287.88 | 3.023.514 |
| **Mali** | 49720.34 | 38618.19 | 3.826.208 | 50317.44 | 37684.61 | 3.743.909 | 50317.44 | 37281.09 | 3.707.285 |
| **Malta** | 50722.13 | 35985.26 | 357.135 | 50430.27 | 35260.38 | 3.500.573 | 50430.27 | 35015.33 | 3.472.926 |
| **Marshall Islands** | 49344.61 | 38749.03 | 3.838.486 | 49143.1 | 38830.81 | 3.841.002 | 49143.1 | 38406.93 | 3.793.866 |
| **Mauritania** | 49652.11 | 37258.57 | 3.710.394 | 50143.36 | 36791.76 | 3.663.292 | 50143.36 | 36454.23 | 3.631.553 |
| **Mauritius** | 52094.23 | 31016.16 | 3.085.452 | 51490.85 | 30271.66 | 3.007.221 | 51490.85 | 29582.59 | 2.937.937 |
| **Mexico** | 51765.05 | 29528.41 | 2.945.412 | 51075.26 | 28254.12 | 2.810.426 | 51075.26 | 27833.77 | 2.767.639 |
| **Micronesia (Federated States of)** | 49055.78 | 38625.57 | 3.849.255 | 49551.12 | 38886.9 | 3.871.136 | 49551.12 | 38673.12 | 3.847.956 |
| **Monaco** | 49386.8 | 32519.49 | 3.225.609 | 50388.27 | 32517.95 | 3.224.688 | 50388.27 | 31966.08 | 3.167.209 |
| **Mongolia** | 49255.05 | 39697.84 | 395.483 | 49140.17 | 39036.14 | 388.693 | 49140.17 | 37768.84 | 3.753.563 |
| **Montenegro** | 42521.03 | 46627.64 | 4.638.542 | 43187.84 | 45926.82 | 4.572.142 | 43187.84 | 44946.32 | 4.464.426 |
| **Morocco** | 48204.22 | 39944.65 | 397.159 | 48817.95 | 39414.5 | 3.916.207 | 48817.95 | 38833.24 | 3.854.415 |
| **Mozambique** | 47842.72 | 42035.13 | 4.147.094 | 49451.58 | 41379.98 | 4.093.354 | 49451.58 | 40222.35 | 3.978.724 |
| **Myanmar** | 51707.43 | 28267.9 | 2.813.645 | 50823.53 | 26814.35 | 2.671.153 | 50823.53 | 26159.19 | 2.606.003 |
| **Namibia** | 49655.45 | 38339.32 | 3.787.757 | 49779.79 | 37753.81 | 3.732.662 | 49779.79 | 37658.07 | 3.719.649 |
| **Nauru** | 50249.94 | 37853.89 | 3.774.797 | 50073.48 | 38209.37 | 380.899 | 50073.48 | 37120.22 | 3.698.436 |
| **Nepal** | 53449.64 | 33071.21 | 3.285.695 | 52892.65 | 34519.37 | 3.437.057 | 52892.65 | 33136.89 | 3.298.515 |
| **Netherlands** | 50963.04 | 32232.57 | 3.206.763 | 51606.98 | 31336.13 | 3.115.178 | 51606.98 | 31449.29 | 3.122.211 |
| **New Zealand** | 52378.78 | 32028.11 | 3.161.801 | 52083.19 | 33803.54 | 3.342.026 | 52083.19 | 30936.91 | 3.056.577 |
| **Nicaragua** | 47538.98 | 35865.81 | 356.629 | 47850.43 | 35294.96 | 3.516.637 | 47850.43 | 34431.93 | 3.429.947 |
| **Niger** | 45602.59 | 43991.82 | 4.373.246 | 50511.92 | 37821.73 | 3.761.922 | 50511.92 | 37534.9 | 3.739.551 |
| **Nigeria** | 53113.76 | 30078.98 | 2.980.108 | 53447.27 | 29770.21 | 2.953.076 | 53447.27 | 29882.04 | 2.967.011 |
| **Niue** | 49561.2 | 37591.76 | 3.740.593 | 49572.37 | 37128.41 | 3.696.146 | 49572.37 | 36681.73 | 3.647.661 |
| **North Macedonia** | 42148.42 | 46657.37 | 4.641.546 | 43788.31 | 45837.73 | 4.567.061 | 43788.31 | 45358.06 | 4.509.193 |
| **Northern Mariana Islands** | 51303.57 | 35296.93 | 3.514.515 | 49732.66 | 35579.45 | 3.535.431 | 49732.66 | 35296 | 3.513.673 |
| **Norway** | 51713.34 | 33827.07 | 3.355.338 | 50999.34 | 35632.27 | 3.534.131 | 50999.34 | 35650.58 | 3.533.042 |
| **Oman** | 49622.04 | 37118.85 | 3.701.593 | 50071.12 | 36666.45 | 3.655.348 | 50071.12 | 36349.05 | 3.619.244 |
| **Pakistan** | 54531.69 | 30441.09 | 3.027.613 | 54763.77 | 30554.02 | 3.040.082 | 54763.77 | 30567.95 | 3.041.117 |
| **Palau** | 50393.17 | 36891.5 | 3.673.409 | 49371.09 | 37015.01 | 3.680.614 | 49371.09 | 36421.3 | 3.614.267 |
| **Palestine** | 48928.43 | 37645.82 | 3.741.991 | 50905.52 | 34982.11 | 3.473.496 | 50905.52 | 35488.3 | 3.521.767 |
| **Panama** | 47258.19 | 37485.52 | 3.731.313 | 47863.57 | 36937.66 | 36.755 | 47863.57 | 35950.06 | 3.576.799 |
| **Papua New Guinea** | 49278.63 | 38872.29 | 3.857.992 | 50001.64 | 38983.91 | 3.870.846 | 50001.64 | 38819.52 | 3.849.255 |
| **Paraguay** | 50638.14 | 31110.72 | 3.101.406 | 51445 | 30523.33 | 3.037.433 | 51.445 | 29937.44 | 2.978.932 |
| **Peru** | 46969.02 | 40258.32 | 4.007.862 | 47192.89 | 39632.98 | 3.950.704 | 47192.89 | 39045.02 | 3.888.872 |
| **Philippines** | 52649.67 | 33984.67 | 3.379.695 | 53456.5 | 32866.93 | 3.272.966 | 53456.5 | 32626.44 | 3.250.135 |
| **Poland** | 49247.05 | 35710.69 | 3.550.618 | 49558.73 | 35201.02 | 3.503.143 | 49558.73 | 35202.27 | 3.498.047 |
| **Portugal** | 50975.82 | 30975.77 | 3.071.283 | 49521.55 | 29410.88 | 2.915.608 | 49521.55 | 29869.38 | 296.453 |
| **Puerto Rico** | 49555.34 | 31665.45 | 3.144.665 | 49574.32 | 31209.66 | 3.101.774 | 49574.32 | 31342.98 | 3.113.973 |
| **Qatar** | 48632.26 | 36015.05 | 3.577.628 | 51908.23 | 36158.7 | 3.603.883 | 51908.23 | 35148.63 | 3.498.888 |
| **Republic of Korea** | 49714.57 | 34368.36 | 3.427.102 | 50323.08 | 33515.12 | 3.338.382 | 50323.08 | 32466.59 | 3.232.813 |
| **Republic of Moldova** | 44844.45 | 44646.75 | 4.434.779 | 45846.27 | 44123.5 | 4.387.556 | 45846.27 | 43286.68 | 4.301.279 |
| **Romania** | 40839.86 | 49567.43 | 4.931.783 | 41755.25 | 48713.63 | 4.846.475 | 41755.25 | 47540.05 | 4.722.456 |
| **Russian Federation** | 49468.04 | 36781.24 | 3.642.691 | 49978.04 | 37775.84 | 374.996 | 49978.04 | 36525.78 | 3.622.616 |
| **Rwanda** | 49701.46 | 38666.07 | 3.808.316 | 50665.92 | 37606.13 | 3.718.571 | 50665.92 | 36847.89 | 3.651.121 |
| **Saint Kitts and Nevis** | 49386.42 | 33445.02 | 3.323.225 | 48992.21 | 33064.9 | 3.284.896 | 48992.21 | 32531.83 | 323.424 |
| **Saint Lucia** | 49509.02 | 34219.59 | 3.400.766 | 49087.77 | 33416.17 | 3.318.272 | 49087.77 | 33487.77 | 3.324.887 |
| **Saint Vincent and the Grenadines** | 47798.7 | 40343.77 | 4.009.237 | 48155.4 | 39739.88 | 3.947.522 | 48155.4 | 39795.61 | 3.948.918 |
| **Samoa** | 49412.17 | 38250.25 | 3.814.758 | 49657.27 | 38004.45 | 378.938 | 49657.27 | 37732.37 | 3.763.814 |
| **San Marino** | 51425.62 | 34496.5 | 3.424.997 | 50856.08 | 33650.45 | 3.333.782 | 50856.08 | 34282 | 3.398.714 |
| **Sao Tome and Principe** | 49682.31 | 37762.02 | 3.762.539 | 49457.99 | 37309.96 | 3.717.758 | 49457.99 | 36029.84 | 3.589.102 |
| **Saudi Arabia** | 47285.52 | 41953.55 | 4.177.847 | 48104.25 | 41067.01 | 4.086.608 | 48104.25 | 40280.79 | 3.997.824 |
| **Senegal** | 46211.6 | 45321.58 | 44.952 | 46341.03 | 44805.8 | 4.445.877 | 46341.03 | 44656.61 | 4.435.952 |
| **Serbia** | 40664.18 | 49769.01 | 4.949.134 | 42338.89 | 62861.96 | 6.248.242 | 42338.89 | 47523.27 | 4.724.378 |
| **Seychelles** | 52491.46 | 30585.59 | 3.056.523 | 51706.84 | 30335.35 | 3.023.863 | 51706.84 | 29275.88 | 2.917.595 |
| **Sierra Leone** | 47879.43 | 40517.57 | 4.006.972 | 48688.9 | 39784.13 | 394.918 | 48688.9 | 39118.07 | 3.892.896 |
| **Singapore** | 50711.47 | 32477.78 | 3.244.844 | 50076.04 | 31823.16 | 3.180.834 | 50076.04 | 31621.27 | 3.152.344 |
| **Slovakia** | 42948.01 | 45995.56 | 4.572.737 | 44294.68 | 44511.29 | 4.429.712 | 44294.68 | 44021.98 | 4.369.043 |
| **Slovenia** | 43033.1 | 43560.48 | 4.322.859 | 45172.65 | 42339.84 | 4.201.675 | 45172.65 | 41400.79 | 4.109.374 |
| **Solomon Islands** | 49407.04 | 39625.89 | 3.946.163 | 48947.75 | 39834.88 | 3.961.812 | 48947.75 | 39112.78 | 3.888.063 |
| **Somalia** | 48985.94 | 40674.99 | 4.025.467 | 48887.94 | 40509.48 | 4.009.754 | 48887.94 | 40301.11 | 3.989.003 |
| **South Africa** | 52159.24 | 32745.49 | 3.234.537 | 52341.47 | 32238.25 | 3.183.377 | 52341.47 | 32511.2 | 3.206.832 |
| **South Sudan** | 50874.86 | 35013.91 | 3.463.309 | 51110.13 | 34554.77 | 3.423.009 | 51110.13 | 34509.21 | 341.445 |
| **Spain** | 50336.32 | 36839.94 | 3.662.509 | 50008.58 | 34374.81 | 3.415.952 | 50008.58 | 35681.18 | 3.542.048 |
| **Sri Lanka** | 51718.93 | 31765.71 | 3.161.907 | 51808.35 | 31326.6 | 3.119.323 | 51808.35 | 30399.26 | 3.029.676 |
| **Sudan** | 48390.04 | 40143.53 | 3.994.337 | 48799.86 | 40200.93 | 4.000.502 | 48799.86 | 38875.92 | 3.870.225 |
| **Suriname** | 48974.34 | 27408.64 | 2.723.513 | 48030.46 | 26459.85 | 2.627.083 | 48030.46 | 26422.88 | 2.625.902 |
| **Sweden** | 50416.39 | 37747.14 | 3.747.403 | 50821.18 | 36971.03 | 3.671.031 | 50821.18 | 36915.35 | 3.665.022 |
| **Switzerland** | 47429.46 | 41624.46 | 4.113.808 | 48361.7 | 41562.2 | 4.108.508 | 48361.7 | 41022.48 | 4.063.639 |
| **Syrian Arab Republic** | 48333.52 | 40024.04 | 3.986.495 | 47316.75 | 39254.68 | 3.911.635 | 47316.75 | 39665.41 | 3.929.819 |
| **Taiwan (Province of China)** | 50685.1 | 26618.69 | 2.658.243 | 50854.18 | 26288.81 | 2.626.156 | 50854.18 | 26565.39 | 2.654.647 |
| **Tajikistan** | 48045.87 | 41338.85 | 4.119.688 | 48179.32 | 40976.5 | 409.261 | 48179.32 | 40095.36 | 3.999.327 |
| **Thailand** | 52388.61 | 31331.94 | 3.119.759 | 51253 | 30643.45 | 3.053.402 | 51.253 | 29542.17 | 2.946.714 |
| **Timor-Leste** | 49719.95 | 38819.2 | 3.832.021 | 50796.78 | 38179.89 | 3.803.313 | 50796.78 | 38035.98 | 3.794.247 |
| **Togo** | 49158.17 | 38100.3 | 377.929 | 49788.98 | 37916 | 3.762.401 | 49788.98 | 37606.74 | 3.737.136 |
| **Tokelau** | 49181.99 | 38490.29 | 3.837.134 | 49723.07 | 38017.86 | 379.086 | 49723.07 | 37365.59 | 3.722.397 |
| **Tonga** | 49633.58 | 38402.43 | 3.830.935 | 49680.42 | 38254.82 | 3.814.079 | 49680.42 | 38010.93 | 3.786.057 |
| **Trinidad and Tobago** | 48947.33 | 33704.23 | 3.350.881 | 49325.79 | 32980.32 | 3.277.405 | 49325.79 | 32645.07 | 3.238.611 |
| **Tunisia** | 47935.22 | 38190.96 | 3.807.304 | 48413.09 | 38653.32 | 3.848.623 | 48413.09 | 37552.47 | 3.732.653 |
| **Turkey** | 45909.59 | 44582.47 | 4.429.275 | 47735.8 | 41040.73 | 4.074.074 | 47735.8 | 40349.43 | 4.008.597 |
| **Turkmenistan** | 48768.26 | 39318.38 | 3.924.623 | 49918.87 | 38283.19 | 381.963 | 49918.87 | 37248.25 | 3.711.782 |
| **Tuvalu** | 49264.91 | 39192.52 | 3.899.161 | 49271.36 | 38758.96 | 3.857.694 | 49271.36 | 38253.07 | 380.972 |
| **Uganda** | 51957.91 | 31321.11 | 3.096.033 | 52250.79 | 30871.71 | 3.063.909 | 52250.79 | 31002.97 | 3.080.828 |
| **Ukraine** | 49704.8 | 36864.12 | 3.659.678 | 49507.93 | 36344.8 | 3.611.706 | 49507.93 | 35872.12 | 3.560.361 |
| **United Arab Emirates** | 50398.32 | 35506.75 | 3.538.049 | 46678.21 | 35787.7 | 3.567.792 | 46678.21 | 35434.95 | 3.514.363 |
| **United Kingdom** | 52094.8 | 33139.13 | 3.274.862 | 52347.75 | 31471.33 | 3.113.697 | 52347.75 | 32777.01 | 3.240.887 |
| **United Republic of Tanzania** | 47224.86 | 42293.87 | 4.175.463 | 49498.14 | 38550.64 | 381.969 | 49498.14 | 38942.3 | 386.413 |
| **United States of America** | 48869.45 | 32162.66 | 3.194.808 | 50177.7 | 31548.68 | 3.134.426 | 50177.7 | 32112.32 | 3.187.735 |
| **United States Virgin Islands** | 51954.25 | 28047.68 | 2.772.812 | 52830.14 | 28040.51 | 2.774.544 | 52830.14 | 28741.36 | 283.636 |
| **Uruguay** | 46803.44 | 41599.28 | 4.131.915 | 47353.67 | 40981.31 | 4.071.695 | 47353.67 | 40150.13 | 3.984.041 |
| **Uzbekistan** | 48317.32 | 40325.54 | 4.016.131 | 49030.21 | 39494.8 | 3.936.264 | 49030.21 | 38799.01 | 3.863.677 |
| **Vanuatu** | 49485.5 | 39048.33 | 3.882.475 | 49387.03 | 39036.26 | 38.786 | 49387.03 | 38821.05 | 3.858.988 |
| **Venezuela (Bolivarian Republic of)** | 47722.07 | 34038.81 | 3.389.237 | 47455.61 | 33236.45 | 3.310.467 | 47455.61 | 33485.04 | 3.329.355 |
| **Viet Nam** | 50332.43 | 35906.82 | 3.590.159 | 51891.73 | 30824.92 | 3.080.814 | 51891.73 | 30684.6 | 3.063.951 |
| **Yemen** | 47649.3 | 40488.78 | 401.144 | 48126.83 | 39848.13 | 3.948.586 | 48126.83 | 40433.43 | 4.001.205 |
| **Zambia** | 51328.55 | 34882.85 | 3.446.694 | 51657.39 | 33840.88 | 3.352.047 | 51657.39 | 33672.74 | 333.538 |
| **Zimbabwe** | 50889.86 | 33052.09 | 3.268.657 | 50292.38 | 34323.38 | 3.395.932 | 50292.38 | 33970 | 3.368.132 |

Rate per 100.00 thousand / Source: Global Burden of Disease (GBD).
